# Effectiveness of Digital Health Interventions to Improve Self-Care in Patients With Chronic Diseases: Systematic Review and Meta-Analysis of Randomized Controlled Trials

**DOI:** 10.2196/88708

**Published:** 2026-06-09

**Authors:** Jessica Longhini, Daniel Pedrotti, Federica Foladori, Melania Stedile, Francesca Stefani, Michela Dal Ben, Alessandro Froner, Marta Proietti Pesci, Stefano Toccoli, Anna Brugnolli

**Affiliations:** 1 Laboratory of Studies and Evidence Based Nursing Universita degli Studi di Padova Dipartimento di Scienze Cardio-Toraco-Vascolari e Sanita pubblica University of Padua Padova, Veneto Italy; 2 Trento Campus of Health Sciences Azienda Sanitaria Universitaria Integrata del Trentino Trento Italy; 3 Interdepartmental Medical Science Centre University of Trento Trento Italy

**Keywords:** chronic disease, digital, e-health, self-care, self-management, technology

## Abstract

**Background:**

Chronic diseases account for most global morbidity and mortality, increasing the need for effective long-term self-care support. Digital health interventions, such as mobile apps, telemonitoring, and connected devices, are increasingly used to promote self-care; yet, their overall effectiveness across chronic conditions remains unclear.

**Objective:**

This systematic review and meta-analysis evaluated whether digital health interventions improve self-care in adults with chronic diseases.

**Methods:**

We searched PubMed, CINAHL, Scopus, and PsycINFO for randomized controlled trials (RCTs; January 1, 2013, to December 31, 2025) that assessed digital health interventions targeting self-care outcomes, as measured with validated instruments, in patients with chronic conditions. Standardized mean differences (SMDs) were pooled using random-effects models, while results not suitable for meta-analysis were synthesized narratively. Risk of bias was assessed with the Cochrane Risk of Bias 2.0 tool for RCTs and certainty of evidence with Grading of Recommendations Assessment, Development and Evaluation.

**Results:**

A total of 55 RCTs involving 5889 participants were included. Most interventions were multicomponent and mainly based on mobile or web-based applications, telemonitoring, connected devices, and text-messaging support. In diabetes, pooled analyses showed little to no clear improvement across self-care domains measured with the Summary of Diabetes Self-Care Activities, including general diet (3 studies), specific diet (3 studies), exercise (5 studies), foot care (5 studies), and glucose monitoring (4 studies), with low to very low certainty of evidence. In heart failure, digital interventions probably improved self-care monitoring measured with the Self-Care of Heart Failure Index (5 studies, 364 participants; SMD=0.49, 95% CI 0.13-0.85; low certainty), whereas effects on self-care maintenance (5 studies) and on self-care measured with the European Heart Failure Self-Care Behaviour Scale (3 studies) were not clearly demonstrated. In other chronic conditions, narrative synthesis suggested possible benefits in some cardiovascular conditions, chronic hepatitis B, epilepsy, and hypertension, while no significant effects were found in chronic obstructive pulmonary disease and multimorbidity, and mixed findings emerged in Parkinson disease. Across 17 studies, medication adherence showed little to no overall improvement (SMD=0.06, 95% CI –0.31 to 0.42, 95% prediction interval –0.98 to 1.09; very low certainty), indicating that future studies could plausibly show either benefit or no effect. Overall, heterogeneity was substantial, and most evidence was of low or very low certainty.

**Conclusions:**

This review is innovative in providing an up-to-date, cross-condition synthesis focused specifically on self-care as a multidimensional outcome, rather than on clinical end points alone or single diseases. The findings suggest that digital health interventions may be more effective for supporting self-care monitoring than for promoting broader behavioral maintenance or medication adherence. Evidence is limited by methodological heterogeneity, small sample sizes, short follow-up periods, and varied outcome measures. Larger designed trials using standardized self-care metrics and equity-focused approaches are needed to clarify effectiveness and guide implementation.

## Introduction

Chronic diseases remain the leading cause of morbidity and mortality worldwide in adults, accounting for over 40 million deaths annually from noncommunicable diseases and imposing an escalating burden on health systems and societies [1]. As global populations age, the prevalence of chronic conditions, such as diabetes, heart failure, chronic obstructive pulmonary disease (COPD), cancer, and chronic kidney disease continues to rise, generating escalating demand for long-term management and self-management support [1-3].

In this scenario, self-care in the adult population has emerged as a cornerstone of chronic disease management to ensure sustainable health systems against the shortage of health professionals and limitations in service accessibility [4]. For the purpose of this review, chronic diseases are defined as long-term conditions requiring ongoing management and self-regulation, such as cardiovascular diseases, diabetes, and chronic respiratory conditions, which are characterized by a persistent need for continuous self-care behaviors. According to the World Health Organization (WHO) [4], self-care refers to “the ability of individuals, families, and communities to promote health, prevent disease, maintain health, and cope with illness and disability, with or without the support of a health care provider.” Building on this, Riegel’s middle-range theory of self-care of chronic illness conceptualizes self-care as a multidimensional process encompassing maintenance (health-promoting behaviors, such as diet, physical activity, smoking, alcohol consumption, mental health, medication adherence, and engagement with health care services), monitoring (recognition and interpretation of symptoms, tracking changes in physical or psychological status, and awareness of early warning signs), and management (decision-making and action in response to symptoms, including medication adjustment, seeking professional support, implementing coping strategies, and modifying daily activities) [5,6]. However, despite this well-established theoretical framework, the measurement of self-care remains highly heterogeneous across studies, with different instruments capturing distinct dimensions of the construct, thus limiting comparability and synthesis of evidence. In particular, medication adherence is among the most extensively investigated self-care behaviors across chronic conditions, given the challenges associated with promoting this competence and its substantial impact on hard clinical outcomes. Indeed, evidence consistently demonstrates that adequate self-care in chronic conditions, including medication adherence, is associated with improved quality of life, reduced hospitalizations, and decreased mortality [7,8]. Nevertheless, previous studies and reviews have reported inconsistent effects of interventions aimed at improving self-care, likely due to variability in intervention components, outcome measures, and methodological quality, highlighting the need for more rigorous and comprehensive syntheses.

Over the past decade, particularly during and after the COVID-19 pandemic, digital health interventions have played a pivotal role in supporting adult patients with chronic conditions in self-care and ensuring continuity of chronic disease management when in-person services were disrupted [9,10]. Digital health interventions encompass telemedicine, mobile health (mHealth), remote monitoring, big data, artificial intelligence, and other technology-driven tools to improve health outcomes [10]. However, the available synthesized evidence remains only partially informative regarding their effectiveness in improving self-care. Most systematic reviews have predominantly focused on clinical and service-oriented outcomes, such as hospitalizations, mortality, symptom severity, and physiological parameters [11-14], while rarely identifying self-care as a primary end point. Even when self-care related outcomes are considered, they are often operationalized through proxy constructs such as self-efficacy or focused on a single behavior, such as physical activity or diet adherence [14,15].

When focusing on self-care, available systematic reviews are largely disease-specific. In heart failure, telemonitoring interventions may improve self-care behaviors, although findings remain inconsistent [16]. In dementia, digital technologies have shown potential to support autonomy and self-management, but with heterogeneous and low-certainty evidence [12,17]. Digital interventions have also shown promise in atopic dermatitis and cancer care, although findings remain context-specific [18,19]. More recent trials and reviews continue to expand the field, showing benefits of digital self-care interventions across conditions such as cardiovascular disease, diabetes, Parkinson disease, asthma, multimorbidity, and COPD [14,15,20,21].

However, considerable variability persists in both outcomes and intervention design. Existing evidence is often limited to specific modalities, such as mobile apps and telemonitoring, thereby overlooking the rapid evolution and increasing diversity of newer digital technologies highlighted in recent literature [15]. Moreover, most studies remain focused on single diseases, even though individuals with chronic conditions commonly experience multimorbidity rather than isolated conditions. Two reviews were conducted on self-care and disease management in chronic disease. One was limited to studies published up to 2020, focused on eHealth, and included only 3 chronic conditions, thereby not reflecting the rapid evolution and diversification of digital health technologies [22]. A recent scoping review mapped the landscape of digital health interventions for chronic disease management. However, by design, it provided a narrative and nonquantitative synthesis, limiting the ability to draw definitive conclusions on effectiveness [15].

Taken together, the literature remains insufficient due to fragmentation across diseases, heterogeneous conceptualization and measurement of self-care, and a lack of an evaluation of self-care as a core outcome. Therefore, there is still no systematic review that specifically synthesizes the effectiveness of digital health interventions for self-care across chronic conditions, while incorporating a wide spectrum of contemporary digital technologies. Addressing this gap is crucial because self-care is a key determinant of long-term outcomes, adherence, and the sustainability of health systems. Clarifying whether, for whom, and under what conditions digital interventions strengthen self-care will inform clinical decision-making, policymakers, and scalable implementation.

The aim of this systematic review and meta-analysis was therefore to evaluate the effectiveness of global digital health interventions in improving self-care among adults with chronic diseases.

## Methods

### Design

This systematic review and meta-analysis were conducted according to the Cochrane Handbook for Systematic Reviews of Interventions [23] and reported following the PRISMA (Preferred Reporting Items for Systematic Reviews and Meta-Analyses) [24] (Multimedia Appendix 1) and PRISMA-S (Preferred Reporting Items for Systematic Reviews and Meta-Analyses literature search extension) [25] guidelines. The protocol was registered in PROSPERO (International Prospective Register of Systematic Reviews; CRD42023479314). No deviations from the predefined protocol occurred.

### Eligibility Criteria

We included studies based on the following criteria: (1) randomized controlled trials (RCTs); (2) adults aged ≥18 years with chronic diseases, defined here as long-lasting conditions requiring ongoing management over time, typically characterized by slow progression and the need for sustained self-care activities; (3) participants recruited in any health care or community setting; (4) “digital health interventions for person,” according to the WHO framework, defined as “the capabilities of digital technology that can be implemented to achieve objectives that are targeted toward persons,” including members of the public who are potential or current users of health services and caregivers [10]. Eligible interventions included mHealth and eHealth approaches (eg, telemonitoring, video consultations, mobile apps, and electronic health records), software-based solution, and emerging technologies (eg, artificial intelligence, big data, and genomics) compared with any type of control condition; (5) self-care abilities, including treatment adherence, measured using validated quantitative questionnaire or instruments; and (6) studies published in English. Studies focusing on patients with cancer, mental illness, pregnant women, or in prisons were excluded.

### Literature Search and Study Selection

We searched 4 electronic databases, PubMed (via PubMed), CINAHL (EBSCOhost), Scopus (Elsevier), and PsycINFO (EBSCOhost). We included studies published between January 1, 2013, and December 31, 2025, following the release of 2 key international and European policy documents on digital health in 2012 [26,27]. The search strings are reported in Multimedia Appendix 2. These were developed iteratively based on preliminary scoping searches and refined in consultation with an expert librarian. Additionally, the reference lists of included trials, gray literature, and registries were screened for any additional eligible studies.

All retrieved records were imported into Rayyan (Rayyan Systems Inc), an AI-assisted tool for systematic reviews, which was used to remove duplicates and manage the screening process. Titles and abstracts were independently screened by 2 reviewers (JL and FF). Full texts of potentially eligible studies were assessed independently by 2 reviewers. In this second step, the level of agreement was Cohen κ=0.83 (95% CI 0.71-0.93), demonstrating good interrater reliability. Disagreements were resolved by discussion or consultation with a third reviewer (DP).

### Data Extraction

Two reviewers (JL and FF) independently extracted the following data using a standardized Excel form, which was piloted in 2 studies, after the screening was concluded. The data were authors, year, objectives, design, country, intervention, comparator, setting, participant characteristics, and outcomes (quantitative and narrative data), including follow-up duration and measurement instruments. No disagreements were detected. We contacted 6 corresponding authors of the included studies to request clarification or additional information when data were missing.

### Quality Assessment

The methodological quality of included studies was assessed using the Cochrane Risk of Bias 2.0 tool for RCTs [28]. Discrepancies were resolved by consensus or by consulting a third reviewer. The quality of evidence for each outcome of interest was independently assessed by 2 reviewers using the Grading of Recommendations Assessment, Development and Evaluation (GRADE) system with the GRADEpro software [29]. GRADE is an internationally recognized and widely used framework that provides a transparent, reproducible, and systematic method for rating the certainty of evidence at the outcome level. It considers 5 key domains, risk of bias, inconsistency, indirectness, imprecision, and publication bias, to determine the confidence that the estimated effect is close to the true effect. By evaluating and combining these domains, the overall quality of evidence for each outcome is rated as high, moderate, low, or very low. Language to report confidence in results according to the certainty of evidence was adherent to the Cochrane Handbook [23].

### Data Synthesis and Analysis

The primary outcome was self-care measured with a validated questionnaire. Where sufficient data were available, we performed meta-analyses on comparable outcomes, grouped by outcomes and questionnaires, and homogeneity in digital interventions and conditions. We used standardized mean differences (SMDs, Hedges adjusted *g*) with 95% CIs because different versions of the same questionnaires were used across studies to measure the same outcome. We interpreted an SMD of 0.2, 0.5, and 0.8 as a small, medium, and large effect size, respectively. A random-effects model was used to account for clinical and methodological heterogeneity. Heterogeneity was assessed visually (forest plots) and statistically (*I*^2^ statistic), with thresholds interpreted as follows: 0%-14% negligible, 15%-29% low, 30%-60% moderate, 50%-90% substantial, and 75%-100% considerable heterogeneity [23]. We planned to conduct subgroup analyses if any significant heterogeneity emerged in the pooled results and sensitivity analyses if high-risk-of-bias studies were included in the meta-analysis. Small-study effects were investigated through the visual inspection of the funnel plots and the Egger test when at least 10 studies were available. Prediction intervals were calculated with at least 5 studies and no asymmetry in the funnel plot [23]. No double-counting of participants occurred in the meta-analyses. Each study contributed only independent data, and the same participants were not included more than once within any single analysis. Analysis was performed with RevMan (version 5; Cochrane) and R software (R Foundation for Statistical Computing) with the package *metafor* [30].

When meta-analysis was not feasible (eg, insufficient data and heterogeneous outcome reporting), results were synthesized narratively.

## Results

### Characteristics of Included Studies

**Figure 1:**
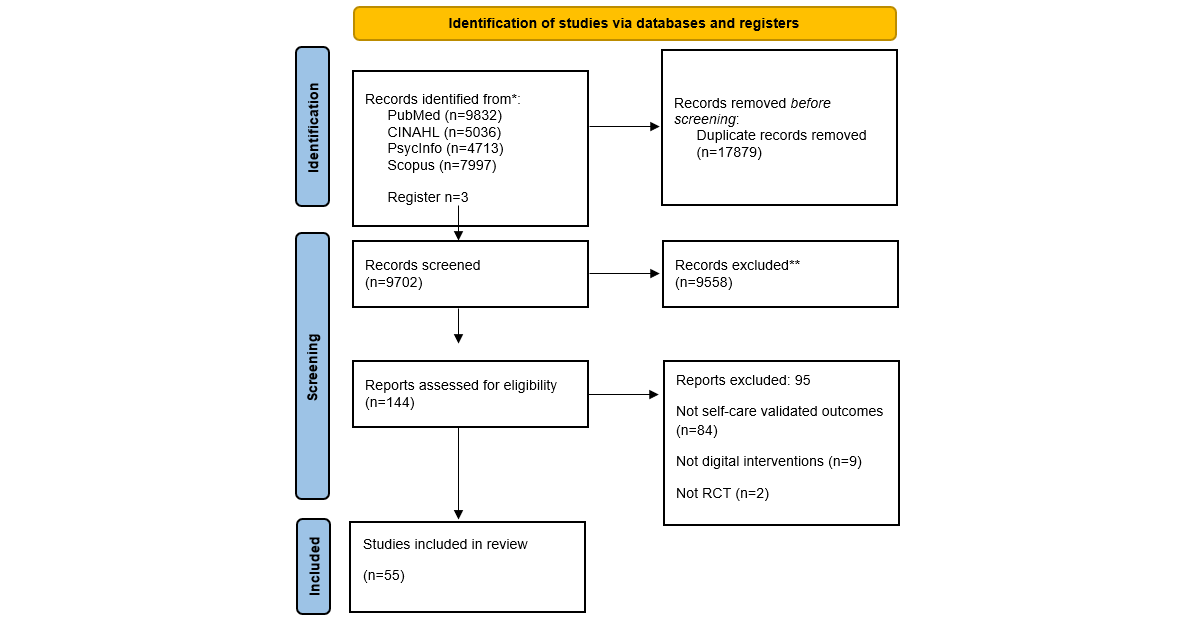
PRISMA (Preferred Reporting Items for Systematic Reviews and Meta-Analyses) flow diagram.

We included 55 RCTs [31-85] (Figure 1), of which almost half (n=26, 47.3%) were published in the past 5 years. The sample sizes ranged from 27 [79] to 1571 [39] (Multimedia Appendix 3 [31-85]). Most studies were conducted in the United States (n=11) [33,48-50,58,63,65,69,79,82,84] and in China (n=10) [43,45,47,61,64,70-72,76,77], followed by Iran [44,53,68], and Australia (n=3) [42,59,75] (Multimedia Appendix 3).

The most common chronic conditions were heart failure (n=13) [37,39,42,44,46,50,55,62,72-75,79], type 1 or type 2 diabetes (n=10) [31,33,35,41,45,47,49,53,59,61], and acute cardiovascular diseases, including myocardial infarction or stroke (n=10) [32,34,36,38,51,56,57,64,65,67], representing the most frequently targeted chronic conditions across the included studies.

Among the included studies, 5 involved participants with multiple chronic diseases [48,58,68,77,78], whereas atrial fibrillation was the primary focus in 4 others [40,52,76,82].

Other less frequent conditions included asthma (n=3) [43,60,84], COPD (n=2) [66,71], hypertension (n=2) [63,85], chronic hepatitis B [54], HIV infection [69], and epilepsy [70].

All studies obtained ethical approval and respected the privacy regulations.

### Characteristics of Interventions

Characteristics of interventions and control care are reported in Table 1 and in Multimedia Appendix 3. Across the 55 included studies, digital health interventions for persons fell within the WHO [10] functional categories of “Targeted communication to persons,” “Personal health tracking,” and “Person-based reporting.”

Mobile or web-based applications were the most frequently used technology (n=39, 70.9%), followed by telemonitoring systems (n=24, 43.6%) and connected medical devices such as blood pressure monitors, glucometers, weight scales, electrocardiogram devices, or activity trackers (n=24, 43.6%). Text-message programs, including SMS text messages, WeChat-based messages, and email/SMS reminders, were used in 18 (32.7%) studies, whereas tablet-based apps were reported in 6 (10.9%) studies.

Overall, interventions were predominantly multicomponent, with the most common configuration combining mobile or web-based applications, telemonitoring, and connected devices (n=12, 21.8%). Stand-alone mobile or web-based applications were used in 10 (18.2%) studies, whereas mobile/web-based application plus text-message programs were reported in 8 (14.5%) studies. Telemonitoring combined with connected devices but without apps was used in 6 (10.9%) studies, and text-message–only interventions were also used in 6 (10.9%) studies.

Interventions can also be characterized according to their main behavior change technique categories [86]. The most common category was prompts/cues, operationalized through reminder messages, push notifications, interactive voice response messages, or automated prompts, which were present in 54 (98.2%) studies. Self-monitoring of behavior or outcomes was also frequent and was usually implemented through apps or connected devices, allowing patients to enter or transmit symptoms, medication use, or physiological parameters; active patient interaction was reported in 44 (80%) studies. Feedback on behavior/biofeedback, often delivered automatically or after clinician review of transmitted data, was commonly paired with alerts for symptoms or high-risk values, which were reported in 33 (60%) studies. A further recurring behavior change technique category was instruction on how to perform the behavior together with information about health consequences, usually delivered through educational modules, videos, app-based content, or structured tele-education, and identifiable in 48 (87.3%) studies. Goal setting, action planning, and behavioral reinforcement were observed in 24 (43.6%) studies, primarily in multicomponent coaching or structured self-management programs. Social support features, such as peer interaction, online discussion boards, caregiver involvement, or shared monitoring functions (eg, “medfriend”), were less common and were identified in 10 (18.2%) studies.

These components were delivered at different levels. Phone calls were incorporated in 21 (38.2%) studies, mainly for follow-up, motivational counseling, or technical support, and face-to-face sessions were included in 12 (21.8%) studies, usually for initial training or reinforcement. All interventions included patient-level components (n=55, 100% studies), such as self-monitoring, reminders, education, and behavioral support. In addition, provider-level involvement, including monitoring of transmitted data, follow-up contacts, or clinical feedback delivered by nurses, pharmacists, physicians, or multidisciplinary teams, was identified in 43 studies (78.2%). In several cases, health care professionals also contributed to treatment adjustment or clinical decision-making based on remotely collected data.

**Table 1:** Characteristics of the interventions.

Study	Mobile app	Tablet	Other devices (eg, BP^a^ and weight)	Text message	Telemonitoring	Reminder/motivational messages	Phone calls	Alerts for symptoms/high risk	Face-to-face sessions	Patient interaction (eg, input parameters)	Conditions
Hoban 2013 [50]	No	No	Yes	No	Yes	No	Yes	Yes	Yes	No	Heart failure
Kirwan 2013 [59]	Yes	No	No	Yes	No	Yes	No	No	No	Yes	Diabetes
Arora 2013 [33]	No	No	No	Yes	No	Yes	No	No	No	No	Diabetes
Heisler 2014 [49]	No	Yes	No	No	No	Yes	Yes	No	Yes	No	Hypertension
Boyne 2014 [37]	No	No	Yes	No	Yes	Yes	No	Yes	No	No	Heart failure
Jahangard-Rafsanjani 2015 [53]	No	No	Yes (glucometer)	No	No	Yes	Yes	No	Yes	Yes	Diabetes
Vuorinen 2014 [73]	Yes	No	Yes	No	Yes	Yes	Yes	Yes	Yes	Yes	Heart failure
Park 2014 [65]	No	No	No	Yes	No	Yes	No	No	No	No	Coronary heart disease
Hägglund 2015 [46]	No	Yes	Yes (weight)	No	Yes	Yes	No	Yes	No	Yes	Heart failure
Pfaeffli Dale 2015 [67]	Yes (web + mobile)	No	Yes (pedometer)	Yes	No	Yes	No	No	No	Yes	Coronary heart disease
Kamal 2015 [57]	No	No	No	Yes	No	Yes	No	No	No	No	Stroke
Jeon 2016 [54]	Yes	No	No	No	No	Yes	No	Yes	No	Yes	Hepatitis B
Akhu-Zaheya 2016 [32]	No	No	No	Yes	No	Yes	No	No	No	No	Cardiovascular diseases
Kim 2016 [58]	Yes	No	Yes (BP monitor)	No	Yes	Yes	No	Yes	No	Yes	Hypertension
Koufopoulos 2016 [60]	Yes (web+mobile)	No	No	No	No	Yes (peer motivational)	No	No	No	Yes	Asthma
Baron 2017 [35]	Yes	No	Yes (BP, glucose)	No	Yes	Yes	Yes	Yes	No	Yes	Diabetes
Melin 2018 [62]	No	Yes	Yes (scale)	No	Yes	Yes	No	Yes	No	No	Heart failure
Desteghe 2018 [40]	No	No	Yes (MEMS^b^)	No	Yes	Yes	Yes	Yes	No	No	Atrial fibrillation
Kamal 2018 [56]	No	No	No	Yes	No	Yes	Yes (IVR^c^)	No	No	Yes	Stroke/MI^d^
Morawski 2018 [63]	Yes	No	No	No	No	Yes	No	No	No	Yes	Hypertension
Schnall 2018 [69]	Yes (web app)	Yes	No	No	No	Yes	No	No	No	Yes	HIV
Agarwal 2019 [31]	Yes	No	No	No	No	Yes	No	No	No	Yes	Hypertension
Sun 2019 [72]	Yes	No	No	Yes (WeChat)	Yes	Yes	Yes	Yes	Yes	Yes	Heart failure
Park 2020 [66]	Yes	No	Yes (pedometer)	Yes	No	Yes	Yes	Yes	Yes	Yes	COPD^e^
Stamenova 2020 [71]	Yes	No	Yes (BP and weight)	No	Yes	Yes	No	Yes	No	Yes	COPD
Ding 2020 [42]	No	No	Yes (scale)	No	Yes	Yes	Yes	Yes	No	Yes	Heart failure
Si 2020 [70]	Yes	No	No	No	Yes	Yes	No	Yes	No	Yes	Epilepsy
Wonggom 2020 [75]	Yes (tablet)	Yes	No	No	No	Yes	No	No	Yes	Yes	Heart failure
Dincer 2020 [41]	Yes	No	No	No	No	Yes	Yes	Yes	Yes	Yes	Diabetes
Hong 2021 [51]	No	No	Yes (BP monitor)	No	Yes	Yes	Yes	Yes	Yes	No	Coronary heart disease
Bruggmann 2021 [38]	Yes	No	Yes (BP, HR^f^, and weight)	No	Yes	Yes	Yes	Yes	No	Yes	HF^g^, COPD, diabetes
Jiang 2021 [55]	Yes	No	Yes (BP and weight)	No	Yes	Yes	Yes	Yes	Yes	Yes	Heart failure
Hsieh 2021 [52]	Yes (web)	Yes	No	No	Yes	Yes	No	Yes	No	Yes	Atrial fibrillation
Ni 2022 [64]	Yes (WeChat)	No	Yes (BP monitor)	Yes	No	Yes	No	Yes	No	Yes	Coronary heart disease
Ware 2022 [74]	Yes	No	Yes (BP, glucose, and weight)	No	Yes	Yes	No	Yes	No	Yes	Hypertension
Han 2023 [47]	Yes	No	Yes (glucometer)	No	Yes	Yes	Yes	Yes	No	Yes	Type 2 diabetes
Poorcheraghi 2023 [68]	Yes	No	No	No	No	Yes	Yes	Yes	No	Yes	Polypharmacy
Deckwart 2023 [39]	No	No	Yes (ECG^h^, BP, weight, and SpO_2_)	No	Yes	Yes	Yes	Yes	No	Yes	Heart failure
Guo 2023 [45]	Yes	No	Yes (glucose sensor)	No	Yes	Yes	Yes	Yes	Yes	Yes	Diabetes
Bernal-Jiménez 2024 [36]	Yes	No	No	No	No	Yes	No	No	No	Yes	Coronary heart disease
FarzanehRad 2024 [44]	No	No	No	Yes	No	Yes	Yes	Yes	No	No	Heart failure
Hartch 2024 [48]	Yes	No	No	No	No	Yes	Yes	Yes	No	Yes	Multiple chronic diseases
Babu 2024 [34]	Yes	No	Yes (BP and glucose)	No	Yes	Yes	No	Yes	No	Yes	Stroke
Ye 2024 [77]	Yes (WeChat)	No	No	Yes	No	Yes	No	No	No	Yes	Diabetes + Hypertension
Xu 2024 [76]	Yes	No	No	No	Yes	Yes	No	Yes	No	Yes	Atrial fibrillation
Erdoğan 2024 [43]	Yes (web-based)	No	No	Yes (email/SMS)	No	Yes	No	No	No	Yes	Asthma
Lee 2024 [61]	Yes	No	No	No	No	Yes	No	Yes	No	Yes	Type 2 diabetes
Hwang 2025 [78]	Yes	No	Yes (BP monitor)	No	Yes	Yes	No	Yes	No	Yes	Hypertension
Keskin 2024 [85]	Yes	No	No	Yes	No	Yes	Yes	Yes	Yes	Yes	Hypertension
Kitsiou 2025 [79]	Yes	No	Yes (BP monitor)	No	Yes	Yes	No	Yes	No	Yes	Hypertension
Lee 2025 [80]	Yes	No	No	Yes	No	Yes	No	No	No	Yes	Type 2 diabetes
Lippke 2025 [81]	Yes	No	No	Yes	No	Yes	No	No	No	Yes	Cardiovascular disease
Magnani 2025 [82]	Yes	No	No	Yes	No	Yes	No	No	No	Yes	Atrial fibrillation
Meyer 2025 [83]	Yes	No	No	No	No	Yes	No	No	No	Yes	Cardiovascular disease
Silberman 2025 [84]	Yes	No	No	Yes	No	Yes	No	No	No	Yes	Cardiovascular disease

^a^BP: blood pressure.

^b^MEMS: Medication Event Monitoring System.

^c^IVR: interactive voice response.

^d^MI: myocardial infarction.

^e^COPD: chronic obstructive pulmonary disease.

^f^HR: heart rate.

^g^HF: heart failure.

^h^ECG: electrocardiogram.

### Risk of Bias

Of the 55 RCTs, 34 (61.8%) were rated as having some concerns, 20 (36.4%) as high risk, and 1 (1.8%) as low risk (Multimedia Appendix 4 [31-85]).

Bias arising from the randomization process: most studies adequately described the random sequence generation and allocation procedures. A total of 40 (72.7%) trials were rated as low risk, while 15 (27.3%) were judged as having some concerns.

Bias due to deviations from intended interventions: in this domain, 35 (63.6%) studies were assessed as low risk, 18 (32.7%) as some concerns, and 2 (3.6%) as high risk.

Bias due to missing outcome data: most trials reported adequate follow-up and outcome data. A total of 37 (67.3%) studies were rated as low risk, 16 (29.1%) as some concerns, and 2 (3.6%) as high risk.

Bias in the measurement of the outcome: the majority of studies were judged as having some concerns in this domain (n=53, 96.4% studies), while 2 (3.6%) studies were rated as high risk.

Bias in selection of the reported result: most studies were judged as low risk (n=39, 70.9% studies), whereas 16 (29.1%) were rated as having some concerns.

### Outcome Measures

#### Overview

The results are categorized by self-care and medication adherence. Within both categories, we reported the results by meta-analysis, and narrative results emerged from studies not pooled in the meta-analysis due to insufficient data or high heterogeneity in the studies’ characteristics.

#### Self-Care Outcomes

##### Self-Care in Diabetes

###### Summary of Diabetes Self-Care Activities

The results are reported according to the most commonly investigated domains, which include general diet, specific diet, exercise, foot care, and glucose monitoring, as specified in the original instrument.

**Figure 2:**
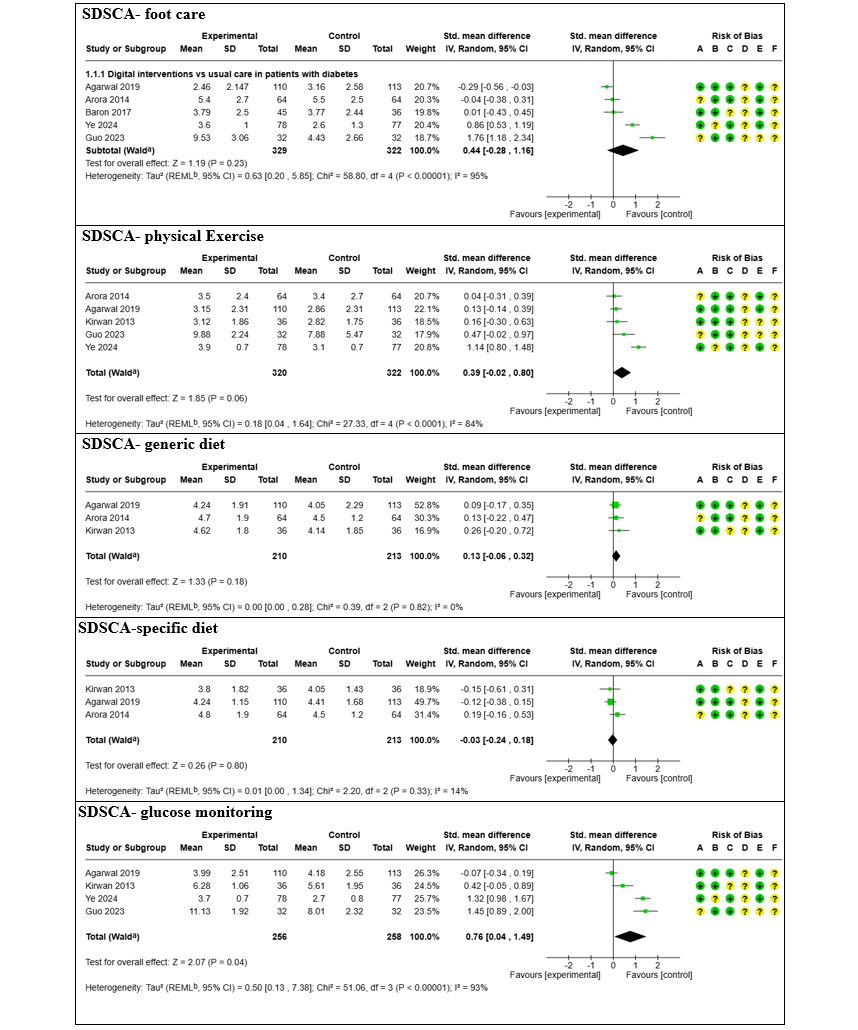
Meta-analysis for the Summary of Diabetes Self-Care Activities (SDSCA) outcomes. HKSJ: Hartung-Knapp-Sidik-Jonkman; REML: restricted maximum likelihood.

Foot care: the pooled estimate from 5 RCTs in patients with diabetes mixing different types of digital interventions [31,33,35,45,77] showed that digital interventions may result in little to no improvement in foot care compared with usual care (SMD=0.44, 95% CI –0.60 to 1.47; *I*^2^=95%; Figure 2 [31,33,35,45,77]), but the evidence is very uncertain. According to the GRADE approach, the certainty of the evidence was rated as very low, downgraded for risk of bias, inconsistency, and imprecision (Table 2).

**Table 2:** Grading of Recommendations Assessment, Development and Evaluation certainty of evidence.

	Certainty assessment									Patients, n			Effect		Certainty
	Studies, n	Study design	Risk of bias	Inconsistency	Indirectness	Imprecision	Other considerations		DHIs^a^		Usual care	SMD^b^ (95% CI)			
SDSCA^c^-foot care	5	RCTs	Serious^d^	Serious^e^	Not serious	Serious^f^	None	329			322	0.44 SD higher (–0.60 to 1.47)		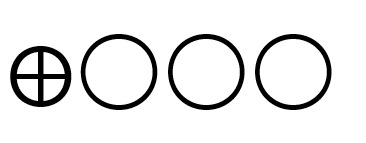 Very low^d^^,e^^,f^	
SDSCA-physical exercise	5	RCTs	Serious^d^	Serious^e^	Not serious	Serious^f^	None	320			322	0.39 SD higher (–0.18 to 0.96)		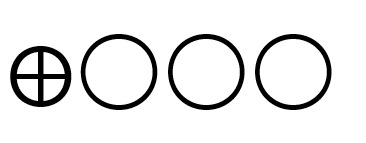 Very low^d^^,e^^,f^	
SDSCA-generic diet	3	RCTs	Serious^d^	Not serious	Not serious	Serious^f^	None	210			213	0.13 SD higher (–0.06 to 0.32)		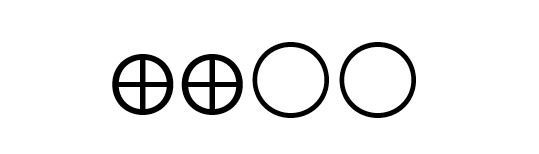 Low^d^^,f^	
SDSCA-specific diet	3	RCTs	Serious^d^	Not serious	Not serious	Serious	None	210			213	0.03 SD lower (–0.47 to 0.42)		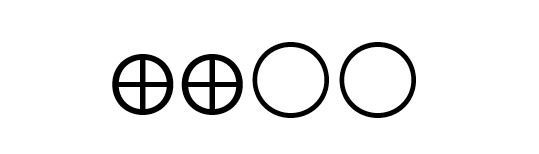 Low^d^	
SDSCA-glucose monitoring	4	RCTs	Serious^d^	Very serious^e^	Not serious	Very serious^f^	None	256			258	0.76 SD higher (–0.40 to 1.93)		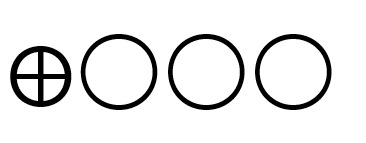 Very low^,^^d^^,e,^^f^	
European Heart Failure Self-Care Behaviour Scale	3	RCTs	Serious^d^	Very serious^e^	Not serious	Very serious^f^	None	1040			1007	–0.18 SD lower (–0.74 to 0.39)		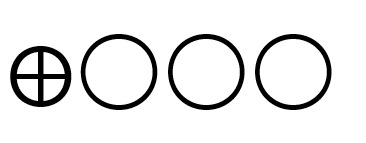 Very low^d,f,g^	
Self-Care of Heart Failure Index-maintenance	5	RCTs	Serious^d^	Very serious^e^	Not serious	Serious^f^	None	208			156	0.48 SD higher (–0.10 to 1.05)		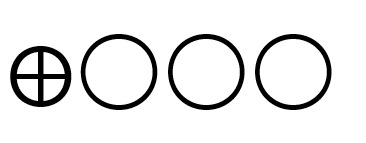 Very low^d,f^	
Self-Care of Heart Failure Index-monitoring	5	RCTs	Serious^d^	Serious^e^	Not serious	Not serious	None	208			156	0.49 SD higher (0.13 to 0.85)		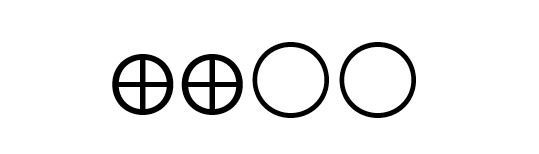 Low^d^^,e^	
Medication adherence	17	RCTs	Serious^d^	Serious^e^	Not serious	Serious^f^	None	1372			1383	0.06 SD higher (–0.31 to 0.42)		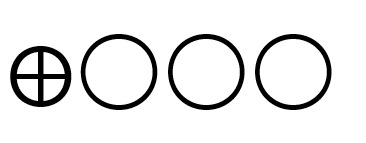 Very low^d^^,f^	

^a^DHI: digital health intervention.

^b^SMD: standardized mean difference.

^c^SDSCA: Summary of Diabetes Self-Care Activities.

^d^Some concerns were reported in different domains across studies.

^e^Inconsistency was partially explained by study location.

^f^The 95% CI includes no meaningful benefit and no effect or arm.

^g^No reasons were identified to explain the statistical heterogeneity.

In subgroup analysis (Multimedia Appendix 5 [31,33,35,45,59,77]), when considering studies conducted in high-income countries (United States, United Kingdom, and Canada), statistical heterogeneity markedly decreased (*I*^2^=14%) and the pooled effect continued to show no meaningful improvement in self-care (SMD=–0.15, 95% CI –0.57 to 0.27). Studies conducted in China showed no meaningful improvement in self-care, and the statistical heterogeneity remained high (SMD=1.27, 95% CI –4.42 to 6.97; *I*^2^=86%). The sensitivity analyses yielded results consistent with the primary analysis.

Physical exercise: the pooled estimate from 5 RCTs mixing different types of digital interventions [31,33,45,59,77] showed that digital interventions may result in a small improvement in physical exercise self-care compared with usual or augmented usual care (SMD=0.39, 95% CI –0.18 to 0.96; *I*^2^=84%; Figure 2). According to the GRADE approach, the certainty of the evidence was rated as low, downgraded for risk of bias and imprecision (Table 2).

In subgroup analysis (Multimedia Appendix 5), studies conducted in high-income countries (United States, United Kingdom, and Canada) showed no meaningful improvement in physical exercise self-care (SMD=0.11, 95% CI –0.03 to 0.25; *I*^2^=0%), as well as studies conducted in China (SMD=0.83, 95% CI –3.38 to 5.04; *I*^2^=79%). The sensitivity analyses yielded results consistent with the primary analysis.

Generic diet: the pooled estimate of 3 studies mixing different types of digital interventions [31,33,59] showed that digital interventions may result in little to no improvement in general dietary self-care compared with usual or augmented usual care (SMD=0.13, 95% CI –0.06 to 0.32; *I*^2^=0%; Figure 2). According to the GRADE approach, the certainty of the evidence was rated as low, downgraded for risk of bias and imprecision (Table 2).

Specific diet: the pooled estimate of 3 studies mixing different types of digital interventions [31,33,59] showed that digital interventions may result in little to no difference in specific dietary self-care compared with usual or augmented usual care (SMD=–0.03, 95% CI –0.47 to 0.42; *I*^2^=14%; Figure 2). According to the GRADE approach, the certainty of the evidence was rated as low, downgraded for risk of bias and imprecision (Table 2).

Glucose monitoring: the pooled estimate from 4 RCTs mixing different types of digital interventions [31,45,59,77] showed that digital interventions may result in little to no difference in glucose monitoring self-care compared with usual care (SMD=0.76, 95% CI –0.40 to 1.93; *I*^2^=93%; Figure 2), but the evidence is very uncertain. According to the GRADE approach, the certainty of the evidence was rated as very low, downgraded for risk of bias, inconsistency, and imprecision (Table 2).

In subgroup analysis (Multimedia Appendix 5), studies conducted in high-income countries (United States and United Kingdom) showed no meaningful improvement in glucose monitoring self-care (SMD=0.13, 95% CI –2.98 to 3.25; *I*^2^=70%), whereas studies conducted in China showed a large and statistically significant effect favoring digital interventions (SMD=1.36, 95% CI 0.66 to 2.06; *I*^2^=0%). The difference between subgroups was statistically significant (*P*<.001). The sensitivity analyses yielded results consistent with the primary analysis.

###### Narrative Results

Across studies not included in the meta-analysis (Multimedia Appendix 6 [41,45,53,61,67]), digital education and monitoring tools were generally associated with improvements in adherence to dietary recommendations, physical activity, and glucose monitoring. Evidence from mHealth and tele-education programs suggests a positive effect on dietary behaviors and overall self-care, as reflected by improvements in Summary of Diabetes Self-Care Activities (SDSCA) scores across several studies [45,61,77]. Similarly, pharmacist-supported digital education was associated with improvements in broader self-care domains, including diet, glucose monitoring, and foot care [53]. Interventions specifically targeting foot care through mobile apps also demonstrated potential benefits for self-care behaviors [41].

##### Self-Care in Heart Failure

###### European Heart Failure Self-Care Behaviour Scale

**Figure 3:**
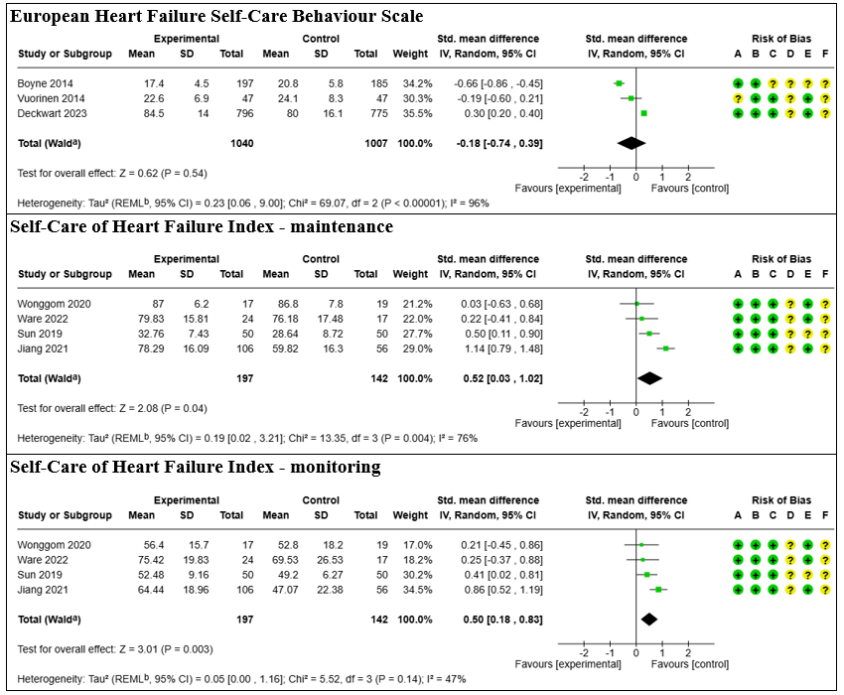
Meta-analysis for the Self-care in heart failure outcomes. HKSJ: Hartung-Knapp-Sidik-Jonkman; REML: restricted maximum likelihood.

The pooled estimate from 3 studies mixing different types of digital interventions [37,39,73] showed that digital interventions may result in little to no difference in self-care compared with usual or augmented usual care, but the evidence is very uncertain (SMD=–0.18, 95% CI –1.39 to 1.03; *I*^2^=96%; Figure 3 [37,39,73]). According to the GRADE approach, the certainty of the evidence was rated as very low, downgraded for risk of bias, inconsistency, and imprecision (Table 2).

###### Self-Care of Heart Failure Index

Maintenance: the overall pooled analysis from 5 studies mixing different types of digital interventions based on an application [55,72,74,75,79] that the experimental intervention may result in little to no improvement in the outcome when compared with the control (SMD=0.48, 95% CI –0.10 to 1.05; *P*=.08; Figure 3) with moderate heterogeneity (*I*^2^=70%; *P*=.005). According to the GRADE approach, the certainty of the evidence was rated as very low due to inconsistency, imprecision, and risk of bias (Table 2).

Subgroup analyses (Multimedia Appendix 5 [55,72,74,75,79]) were conducted according to study location. Among studies performed in China [55,72] (n=262), the SMD showed no significant effect (SMD=0.83, 95% CI –3.19 to 4.85; *P*=.009), with high heterogeneity (*I*^2^=82%), as well as studies conducted in Canada-Australia [74,75,79] (n=102; SMD=0.14, 95% CI –0.12 to 0.40; *P*=.15) but with no heterogeneity (*I*^2^=0%). The sensitivity analyses yielded results consistent with the primary analysis.

Monitoring: the overall pooled analysis from 5 studies mixing different type of digital interventions based on an application [55,72,74,75,79] showed that the experimental intervention probably results in a moderate improvement in self-care monitoring when compared with the control (SMD=0.49, 95% CI 0.13-0.85; *P*=.02; Figure 3) with moderate heterogeneity (*I*^2^=38%; *P*=.21). According to the GRADE approach, the certainty of the evidence was rated as low, downgraded due to inconsistency and risk of bias (Table 2).

Subgroup analyses (Multimedia Appendix 5) were conducted according to study location. Among studies conducted in China [55,72], the SMD showed no statistically significant effect (SMD=0.65, 95% CI –2.14 to 3.45; *P*=.21) with substantial heterogeneity (*I*^2^=64%; *P*=.10). In contrast, in the studies conducted in Canada-Australia [74,75], the SMD favored the experimental intervention, showing a moderate and statistically significant effect (SMD=0.25, 95% CI 0.12-0.38; *P*=.01) and no heterogeneity (*I*^2^=0%; *P*=.98). The test for subgroup differences was not statistically significant (*P*=.08). The sensitivity analyses yielded results consistent with the primary analysis.

###### Narrative Results

Contrasting results emerged from studies not included in the meta-analysis (Multimedia Appendix 6 [37,46,50,62,79]).

Interventions combining daily monitoring with clinician oversight, often involving nurse-led review and feedback, were generally associated with better self-care performance as measured by the European Heart Failure Self-Care Behaviour Scale (EHFScB)-9 and related instruments [46,62]. Positive changes in specific behavioral domains, such as physical activity, were also observed in some interventions, including telemonitoring combined with home-based support, although effects were not consistent across all follow-up time points [50].

Similarly, programs integrating interactive educational components, automated alerts, and patient-provider communication reported improvements in compliance-related outcomes [37]. Conversely, a recent mHealth intervention integrating remote monitoring, education, and motivational messaging did not demonstrate meaningful differences in Self-Care of Heart Failure Index (SCHFI) self-care management compared with usual care [79].

##### Self-Care in Other Chronic Conditions

Other studies investigated digital interventions in different conditions (Multimedia Appendix 6 [51,54,70,78,80,81]).

Overall, digital interventions incorporating education, monitoring, and communication components showed heterogeneous effects on self-care outcomes depending on the condition and measurement tool. In cardiovascular conditions, interventions combining telemonitoring with educational support and follow-up were associated with improvements in self-care as measured by the Partners in Health Scale [51]. Similarly, app-based self-management interventions incorporating educational modules, symptom tracking, and clinician communication in other conditions, including chronic hepatitis B, epilepsy, and hypertension, were linked to positive changes in self-care [54,70,81]. In contrast, studies conducted in patients with COPD generally reported no meaningful differences in self-care outcomes when using mobile apps or telemonitoring systems, including those integrating remote monitoring and clinician support [66,71].

Recent studies using digital health coaching programs and mobile apps reported mixed findings across domains of the Self-Care of Chronic Illness Inventory. While some interventions suggested improvements in specific components, such as self-care maintenance and monitoring in Parkinson disease [80], others did not show statistically significant differences across self-care domains in patients with multiple chronic conditions [78].

#### Medication Adherence

##### Overview

**Figure 4:**
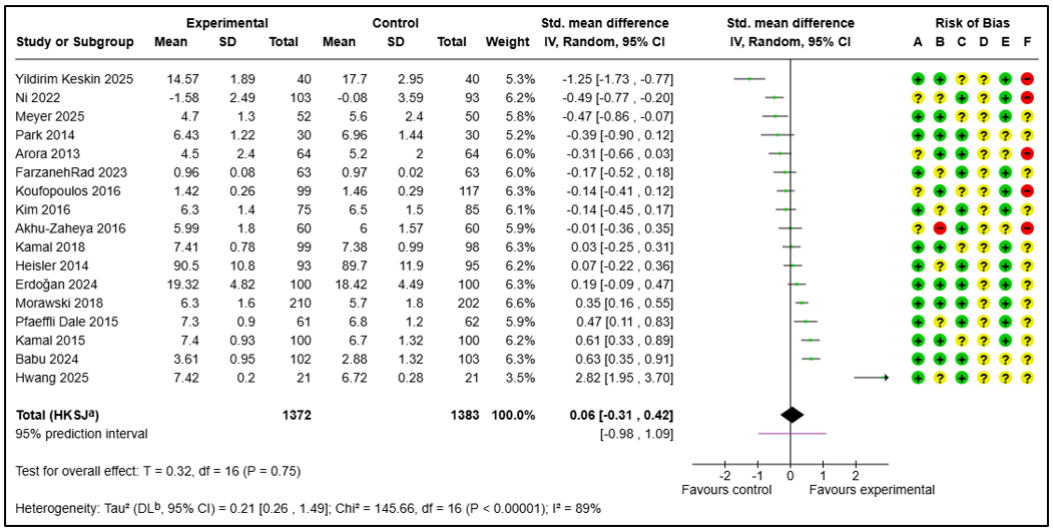
Meta-analysis for the Medication adherence outcome. HKSJ: Hartung-Knapp-Sidik-Jonkman; DL: DerSimonian-Laird.

The overall pooled analysis of 17 studies showed that digital interventions may result in little to no improvement in medication adherence compared with usual or augmented usual care (SMD=0.06, 95% CI –0.31 to 0.42; *I*^2^=89%; Figure 4 [32-34,43,44,49,56-58,60,63-65,67]), but the evidence is very uncertain. The 95% prediction interval (–0.98 to 1.09) indicated that future studies could plausibly show either benefit or no effect. According to the GRADE approach, the certainty of the evidence was rated as very low, downgraded for risk of bias, inconsistency, and imprecision (Table 2).

Subgroup analyses did not meaningfully alter these findings. Grouping studies by type of intervention (text messaging/reminders vs multicomponent interventions) did not reduce heterogeneity and confirmed the absence of a significant effect. Grouping by country income level slightly increased the heterogeneity among studies in both the high-income and low-medium income country groups and still showed no significant improvement in adherence. The sensitivity analyses yielded results consistent with the primary analysis.

##### Narrative Results

Several studies not included in the meta-analysis provide further insights (Multimedia Appendix 6). Across studies using the Morisky Medication Adherence Scale (MMAS-4 and MMAS-8), interventions combining remote monitoring, educational content, and personalized feedback are generally associated with higher adherence levels across multiple conditions, including atrial fibrillation, multimorbidity, and diabetes [40,53,68]. mHealth apps supporting medication management and patient-provider communication also suggested beneficial effects on adherence in atrial fibrillation [76], as did digitally supported behavioral interventions in coronary heart disease [36]. Studies using alternative adherence measures, including the Medication Adherence Rating Scale (MARS) and the Center for Adherence Support Evaluation Adherence Index, also reported positive changes in adherence with interventions based on web platforms, interactive applications, and tailored digital feedback in patients with atrial fibrillation and HIV [52,69]. In contrast, a smartphone-based intervention incorporating a conversational agent and remote monitoring in patients with atrial fibrillation did not show differences in self-reported nonadherence over follow-up [82]. Findings from studies using the Adherence to Refills and Medication Scale (ARMS) were heterogeneous across conditions. Mobile app–based interventions without intensive support showed no differences in adherence in patients with cardiovascular disease [38], whereas interventions incorporating reminders, feedback, and engagement features reported improved adherence in patients with chronic conditions requiring long-term pharmacotherapy [48]. More recent interventions integrating wearable monitoring, personalized feedback, and behavioral interventions suggested potential benefits for adherence in patients with asthma [84].

## Discussion

### Principal Findings

This systematic review and meta-analysis evaluated the effectiveness of digital health interventions in improving self-care and medication adherence among adults with chronic diseases. A total of 47 RCTs were included, encompassing a wide range of chronic conditions, geographic settings, and digital modalities. Overall, the evidence suggests that digital interventions can support patient self-care and adherence behaviors; however, the effects were heterogeneous and not consistently statistically significant.

For diabetes, all SDSCA domains (foot care, diet, physical exercise, and glucose monitoring) showed small, nonsignificant effects with low to very low certainty, indicating that confidence in these estimates is limited. Notably, subgroup analyses in glucose monitoring revealed significant effects in studies conducted in China compared to those conducted in the United States and the United Kingdom, suggesting that contextual factors, such as program intensity, cultural adaptation, and professional involvement, may influence outcomes. Across studies not included in the meta-analysis, digital interventions, particularly those integrating education, monitoring, and pharmacist or clinician support, tended to show improvements in multiple self-care domains (eg, diet, glucose monitoring, and foot care). However, these studies are likely affected by methodological limitations highlighted in the risk of bias assessment. Our meta-analysis findings are inconsistent with the existing body of evidence, although the results from the narrative synthesis are consistent with those reported by Liu et al [87], who found significant improvements in hemoglobin A_1c_, blood pressure, and diabetes self-management activities, especially in short-term interventions (<6 months) and younger populations, and with Shrivastava et al [88], who observed general improvement trends but limited statistical significance due to small samples and unclear risk of bias.

For heart failure, pooled analyses suggested small to moderate improvements in self-care monitoring when measured with the SCHFI instrument, particularly in studies conducted in Canada, the United States, and Australia, compared to those in China, while little to no effects were found in maintenance, nor on self-care when measured with the EHFScB scale. However, certainty of evidence ranged from very low to low, indicating limited confidence in these estimates and that future evidence is likely to change the direction or magnitude of the observed effects. Our narrative synthesis indicates heterogeneous and inconsistent findings; interventions incorporating clinician oversight and interactive components were associated with improvements in specific self-care behaviors, although these findings should be interpreted cautiously, given the generally high or unclear risk of bias across several domains. The absence of an effect on self-care maintenance likely reflects the current capacity of available technologies to support the detection and monitoring of clinical parameters, while remaining insufficient to effectively influence self-care maintenance, defined as behavioral change in key habits [6], such as physical activity, diet, and medication adherence. Overall, our results highlight the uncertainty of the current evidence reporting mixed findings across studies, suggesting that effectiveness remains uncertain, although interactive, feedback-driven models may offer potential benefits [89,90].

This is also supported by the pooled estimate from 17 RCTs on medication adherence across different conditions, which showed no significant overall effect, with very low certainty, indicating that the true effect is likely to be substantially different from the observed estimate and that confidence in this finding is very limited. This was supported by the wide prediction interval, which indicates substantial variability in effects across different settings, suggesting that future studies may plausibly show benefit, no effect, or even harm. No further evidence emerged from subgroup analyses by intervention type and country. This is supported by the narrative synthesis that showed contrasting results, even though findings are predominantly derived from studies at high or moderate risk of bias, requiring attention in interpreting the results. This contrasts with Lanke et al [21] and Kim et al [20], who found significant improvements, especially when apps included interactive functions, enhanced reminders, or data sharing. Shrivastava et al [88] similarly observed improvement trends but noted that few studies reached significance, largely due to methodological limitations. In stroke, Zeng et al [91] reported significant adherence gains from mHealth apps and messaging interventions.

Differences between our findings and prior meta-analyses may reflect broader inclusion criteria, greater heterogeneity in populations and interventions, and the inclusion of multiple digital modalities (apps, telemonitoring, SMS text messages, and web portals), which may have diluted specific adherence effects. Many included trials were small, short-term, and used heterogeneous adherence measures (MMAS, MARS, ARMS, and Medication Event Monitoring System), which reduced the precision and certainty of the evidence.

In other chronic conditions, narrative findings suggest that multicomponent digital interventions may improve self-care in some diseases, particularly cardiovascular conditions, chronic hepatitis B, epilepsy, and hypertension, where improvements were reported in domains such as symptom monitoring, treatment adherence, and overall self-management. In contrast, no significant effects were observed in COPD, while mixed results emerged for Parkinson disease, where improvements were limited to specific domains such as monitoring and maintenance but not management, and for patients with multimorbidity, among whom no significant changes were reported across self-care domains. However, given that the majority of studies were assessed as having a high or moderate risk of bias, these results should be interpreted with caution.

Overall, the inconsistency in our findings may be explained by broader inclusion criteria, greater heterogeneity in populations and outcomes, and diverse intervention modalities. Our review encompassed multiple chronic conditions and technologies (apps, telemonitoring, SMS text messages, and web portals), which may dilute specific effects on medication adherence. Additionally, many included studies were small, of short duration, or used heterogeneous measurement tools (MMAS, MARS, ARMS, and Medication Event Monitoring System), leading to lower precision and downgraded certainty. Furthermore, despite efforts to ensure methodological consistency, self-care remains a complex and multidimensional construct to measure [92], and its measurement varies substantially across studies. The use of different validated instruments, each capturing distinct dimensions of self-care, may limit comparability and contribute to heterogeneity in the findings.

In terms of clinical implications, this review both corroborates, contrasts, and extends prior literature. It confirms that digital health interventions may hold potential for improving self-care, especially in heart failure and monitoring, while also indicating that their effects are uneven and context-dependent [14,15,20,21]. Specifically, due to inconsistent self-care maintenance results across diabetes, heart failure, and other conditions, it remains necessary to support the implementation of digital health interventions with human components to ensure long-term engagement of the person, such as in-person visits and motivational interviewing [93,94]. Other challenges should also be considered in implementing digital health interventions. Equity and digital health literacy remain critical determinants of effectiveness that are not thoroughly addressed in the included studies. Evidence from Turnbull et al [95] showed that the impact of digital self-care interventions is not uniform across populations. Their review showed that web-based programs can benefit disadvantaged or minority groups (eg, ethnic minorities and individuals with lower socioeconomic status), although digital access, health literacy, and usability continue to pose substantial barriers. Ge et al [96] highlight that digital exclusion among older adults is a multidimensional phenomenon that includes resource, skill, and motivational constraints limiting engagement with digital health interventions. Sociodemographic, functional, and psychological factors, such as limited confidence and technology-related anxiety, also reduce participation [97,98] but were rarely analyzed as moderators in the studies included in this review. Therefore, this systematic review highlights the importance of integrating digital interventions into routine care through structured implementation strategies. These include ensuring education for both patients and professionals, tailoring interventions to individual levels of digital literacy, and embedding digital tools within existing clinical pathways to enhance continuity rather than create parallel workflows. Moreover, co-design approaches and ongoing user engagement should be prioritized to improve usability, acceptability, and adherence [10,93]. Health systems adopting digital self-care solutions should also consider equity-oriented implementation frameworks to prevent the widening of existing disparities and to ensure that innovations benefit all patient populations.

As regards research implications, several priorities emerge. First, future trials should adopt standardized outcome measures to enhance comparability and enable robust meta-analyses. The proliferation of heterogeneous instruments (eg, multiple versions of SDSCA, MARS, or MMAS) hampers synthesis and reduces external validity. Second, longer-term trials are needed to assess the sustainability of intervention effects, as most included studies had follow-up durations shorter than 12 months. Third, equity-focused research is essential. Few RCTs stratified outcomes by socioeconomic status, age, or eHealth literacy, despite evidence that these factors critically determine effectiveness. Fourth, cost-effectiveness analyses are urgently required, given the resource implications of scaling digital health interventions within health systems. Fifth, future studies should investigate advanced technologies, including artificial intelligence, wearable sensors, and adaptive platforms, to determine whether they can deliver more consistent benefits. Finally, there is a need to conduct studies on self-care in chronic diseases grounded in robust theoretical frameworks, such as the middle-range theory [5,6]. Recent versions of measurement instruments incorporate self-care management as a third component, alongside maintenance and monitoring, which is crucial for developing individuals’ ability to manage their health autonomously.

A limitation of this review is the inclusion of self-care outcomes only when measured with a validated instrument to ensure consistency across measures. However, many self-care behaviors can also be evaluated through alternative methods, such as step counts for physical activity or pill counts and prescription records for medication adherence. Future research could therefore expand on these findings by adopting a broader approach to measuring self-care behaviors. In addition, the search was conducted on 4 databases and was limited to articles published in English, which may have led to the exclusion of relevant studies and introduced language and publication bias.

### Conclusion

In conclusion, current evidence suggests that digital health interventions may provide some benefit in self-care monitoring in heart failure, while showing no clear or consistent effects on self-care maintenance or when assessed with alternative instruments. In diabetes, effects across all self-care domains (diet, physical activity, foot care, and glucose monitoring) were small and nonsignificant. In other chronic conditions, results varied. Positive changes were observed in some cardiovascular conditions, chronic hepatitis B, epilepsy, and hypertension, whereas no significant effects were found in COPD and multimorbidity, while mixed results emerged in Parkinson disease. Similarly, no clear overall effect was observed for medication adherence across chronic conditions. Overall, the certainty of the evidence is predominantly low to very low, limiting the ability to draw firm conclusions. Results should therefore be interpreted as suggestive but not definitive, emphasizing the need for larger, methodologically robust trials with standardized outcomes and longer follow-up to clarify the true impact of digital health on self-care and adherence. This review synthesizes evidence across multiple chronic conditions, highlighting that the effects of digital health interventions on self-care remain variable and context-dependent. From a practical perspective, integrating digital tools with human support and adapting interventions to patient characteristics may help enhance their effectiveness in routine care.

## Appendix

Multimedia Appendix 1 PRISMA 2020 expanded checklist.

Multimedia Appendix 2 Search strings.

Multimedia Appendix 3 Characteristics of the studies included.

Multimedia Appendix 4 Risk of bias.

Multimedia Appendix 5 Subgroup meta-analysis.

Multimedia Appendix 6 Studies not included in the meta-analysis.

## Abbreviations

ARMS: Adherence to Refills and Medication Scale
COPD: chronic obstructive pulmonary disease
EHFScB: European Heart Failure Self-Care Behaviour Scale
GRADE: Grading of Recommendations Assessment, Development and Evaluation
MARS: Medication Adherence Rating Scale
mHealth: mobile health
MMAS: Morisky Medication Adherence Scale
PRISMA: Preferred Reporting Items for Systematic Reviews and Meta-Analyses
PRISMA-S: Preferred Reporting Items for Systematic Reviews and Meta-Analyses literature search extension
PROSPERO: International Prospective Register of Systematic Reviews
RCT: randomized controlled trial
SCHFI: Self-Care of Heart Failure Index
SDSCA: Summary of Diabetes Self-Care Activities
SMD: standardized mean difference
WHO: World Health Organization

## Data Availability

All data generated or analyzed during this study are included in this published article.

## References

[1] World Health Organization (2025). Noncommunicable Diseases Progress Monitor 2025.

[2] United Nations (2023). World Social Report 2023: Leaving No One Behind In An Ageing World.

[3] World Health Organization (2024). Integrated Care for Older People (ICOPE): Guidance for Person-Centred Assessment and Pathways in Primary Care.

[4] World Health Organization (2021). WHO Guideline on Self-Care Interventions for Health and Well-Being.

[5] Riegel B, Dickson VV, Faulkner KM (2016). The situation-specific theory of heart failure self-care: revised and updated. J Cardiovasc Nurs.

[6] Riegel B, Jaarsma T, Strömberg A (2012). A middle-range theory of self-care of chronic illness. ANS Adv Nurs Sci.

[7] Riegel B, Westland H, Iovino P, Barelds I, Bruins Slot J, Stawnychy MA, Osokpo O, Tarbi E, Trappenburg JC, Vellone E, Strömberg A, Jaarsma T (2021). Characteristics of self-care interventions for patients with a chronic condition: a scoping review. Int J Nurs Stud.

[8] Lee CS, Westland H, Faulkner KM, Iovino P, Thompson JH, Sexton J, Farry E, Jaarsma T, Riegel B (2022). The effectiveness of self-care interventions in chronic illness: a meta-analysis of randomized controlled trials. Int J Nurs Stud.

[9] Moynihan R, Sanders S, Michaleff ZA, Scott AM, Clark J, To EJ, Jones M, Kitchener E, Fox M, Johansson M, Lang E, Duggan A, Scott I, Albarqouni L (2021). Impact of COVID-19 pandemic on utilisation of healthcare services: a systematic review. BMJ Open.

[10] World Health Organization (2023). Classification of Digital Interventions, Services and Applications in Health.

[11] Ambrosi E, Mezzalira E, Canzan F, Leardini C, Vita G, Marini G, Longhini J (2025). Effectiveness of digital health interventions for chronic conditions management in European primary care settings: systematic review and meta-analysis. Int J Med Inform.

[12] Cornelius G, Hodgson W, Maguire R, Egan K (2025). Wearable technology, smart home systems, and mobile apps for the self‑management of patient outcomes in dementia care: systematic review. J Med Internet Res.

[13] Park Y, Kim EJ, Park S, Lee M (2025). Digital health intervention effect on older adults with chronic diseases living alone: systematic review and meta-analysis of randomized controlled trials. J Med Internet Res.

[14] Zhuang M, Hassan II, W Ahmad WMA, Abdul Kadir A, Liu X, Li F, Gao Y, Guan Y, Song S (2025). Effectiveness of digital health interventions for chronic obstructive pulmonary disease: systematic review and meta-analysis. J Med Internet Res.

[15] Al Mahmud A, Joachim S, Jayaraman PP, Learmonth C, Tyagi S, Forkan ARM, Shuakat M, Wickramasinghe N, Wheeler J, Best S, Trainer A (2026). Digital health interventions to support chronic disease management: systematic scoping review. JMIR Mhealth Uhealth.

[16] Nick JM, Roberts LR, Petersen AB (2021). Effectiveness of telemonitoring on self-care behaviors among community-dwelling adults with heart failure: a quantitative systematic review. JBI Evid Synth.

[17] Neal D, van den Berg F, Planting C, Ettema T, Dijkstra K, Finnema E, Dröes R-M (2021). Can use of digital technologies by people with dementia improve self-management and social participation? A systematic review of effect studies. J Clin Med.

[18] Cherrez-Ojeda I, Robles-Velasco K, Osorio MF, Ormaza Vera A, Sarfraz Z, Sarfraz A, Cherrez A, Cherrez S, Sanchez Caraballo JM (2024). A systematic review and meta-analysis of mobile health applications and telemonitoring in atopic dermatitis self-management. Dermatol Ther (Heidelb).

[19] Lim DSC, Kwok B, Williams P, Koczwara B (2023). The impact of digital technology on self-management in cancer: systematic review. JMIR Cancer.

[20] Kim SK, Park SY, Hwang HR, Moon SH, Park JW (2025). Effectiveness of mobile health intervention in medication adherence: a systematic review and meta-analysis. J Med Syst.

[21] Lanke V, Trimm K, Habib B, Tamblyn R (2025). Evaluating the effectiveness of mobile apps on medication adherence for chronic conditions: systematic review and meta-analysis. J Med Internet Res.

[22] Renzi E, Baccolini V, Migliara G, De Vito C, Gasperini G, Cianciulli A, Marzuillo C, Villari P, Massimi A (2022). The impact of eHealth interventions on the improvement of self-care in chronic patients: an overview of systematic reviews. Life (Basel).

[23] Higgins J, Thomas J, Chandler J, Cumpston M, Li T, Page M (2024). Cochrane Handbook for Systematic Reviews of Interventions version 6.5.

[24] Page MJ, McKenzie JE, Bossuyt PM, Boutron I, Hoffmann TC, Mulrow CD, Shamseer L, Tetzlaff JM, Akl EA, Brennan SE, Chou R, Glanville J, Grimshaw JM, Hróbjartsson A, Lalu MM, Li T, Loder EW, Mayo-Wilson E, McDonald S, McGuinness LA, Stewart LA, Thomas J, Tricco AC, Welch VA, Whiting P, Moher D (2021). The PRISMA 2020 statement: an updated guideline for reporting systematic reviews. BMJ.

[25] Rethlefsen ML, Kirtley S, Waffenschmidt S, Ayala AP, Moher D, Page MJ, Koffel JB, PRISMA-S Group (2021). PRISMA-S: an extension to the PRISMA statement for reporting literature searches in systematic reviews. Syst Rev.

[26] World Health Organization and International Telecommunication Union (2012). National eHealth Strategy Toolkit.

[27] (2012). eHealth Action Plan 2012-2020. European Commission.

[28] Sterne JAC, Savović J, Page MJ, Elbers RG, Blencowe NS, Boutron I, Cates CJ, Cheng H, Corbett MS, Eldridge SM, Emberson JR, Hernán MA, Hopewell S, Hróbjartsson A, Junqueira DR, Jüni P, Kirkham JJ, Lasserson T, Li T, McAleenan A, Reeves BC, Shepperd S, Shrier I, Stewart LA, Tilling K, White IR, Whiting PF, Higgins JPT (2019). RoB 2: a revised tool for assessing risk of bias in randomised trials. BMJ.

[29] Schünemann H, Brożek J, Guyatt G, Oxman A (2013). GRADE Handbook for Grading Quality of Evidence and Strength of Recommendations.

[30] R Core Team (2022). R: a language and environment for statistical computing. R Foundation for Statistical Computing.

[31] Agarwal P, Mukerji G, Desveaux L, Ivers NM, Bhattacharyya O, Hensel JM, Shaw J, Bouck Z, Jamieson T, Onabajo N, Cooper M, Marani H, Jeffs L, Bhatia RS (2019). Mobile app for improved self-management of type 2 diabetes: multicenter pragmatic randomized controlled trial. JMIR Mhealth Uhealth.

[32] Akhu-Zaheya LM, Shiyab WY (2017). The effect of short message system (SMS) reminder on adherence to a healthy diet, medication, and cessation of smoking among adult patients with cardiovascular diseases. Int J Med Inform.

[33] Arora S, Peters AL, Burner E, Lam CN, Menchine M (2014). Trial to examine text message-based mHealth in emergency department patients with diabetes (TExT-MED): a randomized controlled trial. Ann Emerg Med.

[34] Babu V, Sylaja P, Soman B, Varma RP, Ms M, Gl G, Kumar B (2024). A randomized controlled trial of medication adherence and management of risk factors for secondary prevention of stroke (MaMoRS) using a smartphone-based application. Int J Stroke.

[35] Baron JS, Hirani SP, Newman SP (2017). Investigating the behavioural effects of a mobile-phone based home telehealth intervention in people with insulin-requiring diabetes: results of a randomized controlled trial with patient interviews. J Telemed Telecare.

[36] Bernal-Jiménez MÁ, Calle G, Gutiérrez Barrios A, Gheorghe LL, Cruz-Cobo C, Trujillo-Garrido N, Rodríguez-Martín A, Tur JA, Vázquez-García R, Santi-Cano MJ (2024). Effectiveness of an interactive mHealth app (EVITE) in improving lifestyle after a coronary event: randomized controlled trial. JMIR Mhealth Uhealth.

[37] Boyne JJJ, Vrijhoef HJM, Spreeuwenberg M, De Weerd G, Kragten J, Gorgels APM, TEHAF investigators (2014). Effects of tailored telemonitoring on heart failure patients' knowledge, self-care, self-efficacy and adherence: a randomized controlled trial. Eur J Cardiovasc Nurs.

[38] Bruggmann C, Adjedj J, Sardy S, Muller O, Voirol P, Sadeghipour F (2021). Effects of the interactive web-based video "Mon Coeur, Mon BASIC" on drug adherence of patients with myocardial infarction: randomized controlled trial. J Med Internet Res.

[39] Deckwart O, Koehler K, Lezius S, Prescher S, Koehler F, Winkler S (2023). Effects of remote patient management on self-care behaviour in heart failure patients: results from the randomized TIM-HF2 trial. Eur J Cardiovasc Nurs.

[40] Desteghe L, Vijgen J, Koopman P, Dilling-Boer D, Schurmans J, Dendale P, Heidbuchel H (2018). Telemonitoring-based feedback improves adherence to non-vitamin K antagonist oral anticoagulants intake in patients with atrial fibrillation. Eur Heart J.

[41] Dincer B, Bahçecik N (2020). The effect of a mobile application on the foot care of individuals with type 2 diabetes: a randomised controlled study. Health Educ J.

[42] Ding H, Jayasena R, Chen SH, Maiorana A, Dowling A, Layland J, Good N, Karunanithi M, Edwards I (2020). The effects of telemonitoring on patient compliance with self-management recommendations and outcomes of the innovative telemonitoring enhanced care program for chronic heart failure: randomized controlled trial. J Med Internet Res.

[43] Erdoğan EG, Örsal Ö (2024). The effect of web-designed education on medication adherence, asthma control and fatigue in patients with asthma: a randomized controlled trial. Int J Nurs Pract.

[44] FarzanehRad A, Allahbakhshian A, Gholizadeh L, Khalili AF, Hasankhani H (2024). Randomized comparison of the effects of tailored text messaging versus pillbox organizers on medication adherence of heart failure patients. BMC Cardiovasc Disord.

[45] Guo M, Meng F, Guo Q, Bai T, Hong Y, Song F, Ma Y (2023). Effectiveness of mHealth management with an implantable glucose sensor and a mobile application among Chinese adults with type 2 diabetes. J Telemed Telecare.

[46] Hägglund E, Lyngå P, Frie F, Ullman B, Persson H, Melin M, Hagerman I (2015). Patient-centred home-based management of heart failure. Findings from a randomised clinical trial evaluating a tablet computer for self-care, quality of life and effects on knowledge. Scand Cardiovasc J.

[47] Han CY, Zhang J, Ye XM, Lu JP, Jin HY, Xu WW, Wang P, Zhang M (2023). Telemedicine-assisted structured self-monitoring of blood glucose in management of T2DM results of a randomized clinical trial. BMC Med Inform Decis Mak.

[48] Hartch CE, Dietrich MS, Lancaster BJ, Stolldorf DP, Mulvaney SA (2024). Effects of a medication adherence app among medically underserved adults with chronic illness: a randomized controlled trial. J Behav Med.

[49] Heisler M, Choi H, Palmisano G, Mase R, Richardson C, Fagerlin A, Montori VM, Spencer M, An LC (2014). Comparison of community health worker-led diabetes medication decision-making support for low-income Latino and African American adults with diabetes using e-health tools versus print materials: a randomized, controlled trial. Ann Intern Med.

[50] Hoban MB, Fedor M, Reeder S, Chernick M (2013). The effect of telemonitoring at home on quality of life and self-care behaviors of patients with heart failure. Home Healthc Nurse.

[51] Hong PC, Chen KJ, Chang YC, Cheng SM, Chiang HH (2021). Effectiveness of theory-based health information technology interventions on coronary artery disease self-management behavior: a clinical randomized waitlist-controlled trial. J Nurs Scholarsh.

[52] Hsieh HL, Kao CW, Cheng SM, Chang YC (2021). A web-based integrated management program for improving medication adherence and quality of life, and reducing readmission in patients with atrial fibrillation: randomized controlled trial. J Med Internet Res.

[53] Jahangard-Rafsanjani Z, Sarayani A, Nosrati M, Saadat N, Rashidian A, Hadjibabaie M, Ashouri A, Radfar M, Javadi M, Gholami K (2015). Effect of a community pharmacist-delivered diabetes support program for patients receiving specialty medical care: a randomized controlled trial. Diabetes Educ.

[54] Jeon JH (2016). Evaluation of a smartphone application for self-care performance of patients with chronic hepatitis B: a randomized controlled trial. Appl Nurs Res.

[55] Jiang Y, Koh KWL, Ramachandran HJ, Nguyen HD, Lim DS, Tay YK, Shorey S, Wang W (2021). The effectiveness of a nurse-led home-based heart failure self-management programme (the HOM-HEMP) for patients with chronic heart failure: a three-arm stratified randomized controlled trial. Int J Nurs Stud.

[56] Kamal AK, Khalid W, Muqeet A, Jamil A, Farhat K, Gillani SRA, Zulfiqar M, Saif M, Muhammad AA, Zaidi F, Mustafa M, Gowani A, Sharif S, Bokhari SS, Tai J, Rahman N, Sultan FAT, Sayani S, Virani SS (2018). Making prescriptions "talk" to stroke and heart attack survivors to improve adherence: results of a randomized clinical trial (The Talking Rx Study). PLoS One.

[57] Kamal AK, Shaikh Q, Pasha O, Azam I, Islam M, Memon AA, Rehman H, Akram MA, Affan M, Nazir S, Aziz S, Jan M, Andani A, Muqeet A, Ahmed B, Khoja S (2015). A randomized controlled behavioral intervention trial to improve medication adherence in adult stroke patients with prescription tailored Short Messaging Service (SMS)-SMS4Stroke study. BMC Neurol.

[58] Kim JY, Wineinger NE, Steinhubl SR (2016). The influence of wireless self-monitoring program on the relationship between patient activation and health behaviors, medication adherence, and blood pressure levels in hypertensive patients: a substudy of a randomized controlled trial. J Med Internet Res.

[59] Kirwan M, Vandelanotte C, Fenning A, Duncan MJ (2013). Diabetes self-management smartphone application for adults with type 1 diabetes: randomized controlled trial. J Med Internet Res.

[60] Koufopoulos JT, Conner MT, Gardner PH, Kellar I (2016). A web-based and mobile health social support intervention to promote adherence to inhaled asthma medications: randomized controlled trial. J Med Internet Res.

[61] Lee H, Park G, Lee D, Khang AR, Lee MJ (2024). Long-term effects of an automated personalized self-care program for patients with type 2 diabetes. Nurs Health Sci.

[62] Melin M, Hägglund E, Ullman B, Persson H, Hagerman I (2018). Effects of a tablet computer on self-care, quality of life, and knowledge: a randomized clinical trial. J Cardiovasc Nurs.

[63] Morawski K, Ghazinouri R, Krumme A, Lauffenburger JC, Lu Z, Durfee E, Oley L, Lee J, Mohta N, Haff N, Juusola JL, Choudhry NK (2018). Association of a smartphone application with medication adherence and blood pressure control: the MediSAFE-BP randomized clinical trial. JAMA Intern Med.

[64] Ni Z, Wu B, Yang Q, Yan LL, Liu C, Shaw RJ (2022). An mHealth intervention to improve medication adherence and health outcomes among patients with coronary heart disease: randomized controlled trial. J Med Internet Res.

[65] Park LG, Howie-Esquivel J, Chung ML, Dracup K (2014). A text messaging intervention to promote medication adherence for patients with coronary heart disease: a randomized controlled trial. Patient Educ Couns.

[66] Park SK, Bang CH, Lee SH (2020). Evaluating the effect of a smartphone app-based self-management program for people with COPD: a randomized controlled trial. Appl Nurs Res.

[67] Pfaeffli Dale L, Whittaker R, Jiang Y, Stewart R, Rolleston A, Maddison R (2015). Text message and internet support for coronary heart disease self-management: results from the text4heart randomized controlled trial. J Med Internet Res.

[68] Poorcheraghi H, Negarandeh R, Pashaeypoor S, Jorian J (2023). Effect of using a mobile drug management application on medication adherence and hospital readmission among elderly patients with polypharmacy: a randomized controlled trial. BMC Health Serv Res.

[69] Schnall R, Cho H, Mangone A, Pichon A, Jia H (2018). Mobile health technology for improving symptom management in low income persons living with HIV. AIDS Behav.

[70] Si Y, Xiao X, Xia C, Guo J, Hao Q, Mo Q, Niu Y, Sun H (2020). Optimising epilepsy management with a smartphone application: a randomised controlled trial. Med J Aust.

[71] Stamenova V, Liang K, Yang R, Engel K, van Lieshout F, Lalingo E, Cheung A, Erwood A, Radina M, Greenwald A, Agarwal P, Sidhu A, Bhatia RS, Shaw J, Shafai R, Bhattacharyya O (2020). Technology-enabled self-management of chronic obstructive pulmonary disease with or without asynchronous remote monitoring: randomized controlled trial. J Med Internet Res.

[72] Sun J, Zhang ZW, Ma YX, Liu W, Wang CY (2019). Application of self-care based on full-course individualized health education in patients with chronic heart failure and its influencing factors. World J Clin Cases.

[73] Vuorinen AL, Leppänen J, Kaijanranta H, Kulju M, Heliö T, van Gils M, Lähteenmäki J (2014). Use of home telemonitoring to support multidisciplinary care of heart failure patients in Finland: randomized controlled trial. J Med Internet Res.

[74] Ware P, Shah A, Ross HJ, Logan AG, Segal P, Cafazzo JA, Szacun-Shimizu K, Resnick M, Vattaparambil T, Seto E (2022). Challenges of telemonitoring programs for complex chronic conditions: randomized controlled trial with an embedded qualitative study. J Med Internet Res.

[75] Wonggom P, Nolan P, Clark RA, Barry T, Burdeniuk C, Nesbitt K, O'Toole K, Du H (2020). Effectiveness of an avatar educational application for improving heart failure patients' knowledge and self-care behaviors: a pragmatic randomized controlled trial. J Adv Nurs.

[76] Xu W, Huang X, Lin Q, Wu T, Guan C, Lv M, Hu W, Dai H, Chen P, Li M, Zhang F, Zhang J (2024). Application of Alfalfa app in the management of oral anticoagulation in patients with atrial fibrillation: a multicenter randomized controlled trial. BMC Med Inform Decis Mak.

[77] Ye H, Lin L, Zhong D, Chen P, He X, Luo Z, Chen P (2024). The impact of telehealth education on self-management in patients with coexisting type 2 diabetes mellitus and hypertension: a 26-week randomized controlled trial. J Endocrinol Invest.

[78] Hwang M, Lee S, Park GE, Park Y (2025). Effectiveness of a digital health coaching self-management program for older adults living alone with multiple chronic conditions: a randomized controlled trial. Geriatr Nurs.

[79] Kitsiou S, Gerber BS, Buchholz SW, Kansal MM, Sun J, Pressler SJ (2025). Patient-centered mHealth intervention to improve self-care in patients with chronic heart failure: phase 1 randomized controlled trial. J Med Internet Res.

[80] Lee J, Yoo S, Kim Y, Kim E, Park H, Sohn YH, Kim YJ, Chung SJ, Baik K, Kim K, Yoo J (2025). Effect of the Yon PD app on the management of self-care in people with Parkinson disease: randomized controlled trial. J Med Internet Res.

[81] Lippke S, Korte L, Kumar VA, Fach A, Ratz T (2025). Health behavior and disease self-management indicators in patients with cardiovascular diseases using a health app: findings from an RCT. AIMS Public Health.

[82] Magnani JW, Lalama CM, Abebe KZ, Ferry D, Rollman BL, Lancet MQ, Kimani E, Ólafsson S, Bickmore T, Paasche-Orlow MK (2025). A mobile relational agent to enhance atrial fibrillation self-care: primary and secondary outcomes of a randomized controlled trial. Am Heart J.

[83] Meyer B, Riepenhausen A, Betz LT, Jauch-Chara K, Reshetnik A (2025). Internet-based digital intervention to support the self-management of hypertension compared to usual care: results of the HALCYON randomized controlled trial. BMC Cardiovasc Disord.

[84] Silberman J, Sarlati S, Harris B, Lenyoun H, Kaur M, Wagner BG, Bokhari W, Boushey H, Chesnutt A, Sitts K, Zhu P, Willey VJ, Fuentes E, LeKrey M, Alger BL, Muscioni G, Bianchi MT, Bota DA, Taylor TH, Evans M, Amin AN, Stark D, Montanari C, Perry JS, Vian C, Patel M, Poe W, Lee RA (2025). A digital asthma self-management program for adults: a randomized clinical trial. JAMA Netw Open.

[85] Yildirim Keskin A, Özpancar Şolpan N, Değirmenci H (2025). The effect of mobile application follow-up on treatment compliance and self-care management in patients with hypertension: randomized controlled trial. Public Health Nurs.

[86] Michie S, Richardson M, Johnston M, Abraham C, Francis J, Hardeman W, Eccles MP, Cane J, Wood CE (2013). The behavior change technique taxonomy (v1) of 93 hierarchically clustered techniques: building an international consensus for the reporting of behavior change interventions. Ann Behav Med.

[87] Liu F, Li J, Li X, Yang Z, Wang W, Zhao L, Wu T, Huang C, Xu Y (2024). Efficacy of telemedicine intervention in the self-management of patients with type 2 diabetes: a systematic review and meta-analysis. Front Public Health.

[88] Shrivastava TP, Goswami S, Gupta R, Goyal RK (2023). Mobile app interventions to improve medication adherence among type 2 diabetes mellitus patients: a systematic review of clinical trials. J Diabetes Sci Technol.

[89] Lu H, Xu S, Yang J, Zhou Y, Gu Z (2026). Effect of telemedicine on self-care in patients with heart failure: a meta-analysis of randomized controlled trials. Eur J Cardiovasc Nurs.

[90] Ni YX, Liu XH, He L, Wen Y, You GY (2024). Mobile application-based interventions for people with heart failure: a systematic review and meta-analysis. J Nurs Manag.

[91] Zeng Z, Wu T, Lv M, Qian J, Chen M, Fang Z, Jiang S, Zhang J (2022). Impact of mobile health and telehealth technology on medication adherence of stroke patients: a systematic review and meta-analysis of randomized controlled trials. Int J Clin Pharm.

[92] Riegel B, De Maria M, Barbaranelli C, Luciani M, Ausili D, Dickson VV, Jaarsma T, Matarese M, Stromberg A, Vellone E (2024). Measuring self-care: a description of the family of disease-specific and generic instruments based on the theory of self-care of chronic illness. J Cardiovasc Nurs.

[93] Eiselt AK, Kirkendall S, Xiong E, Langner D, Goldfarb M (2025). Achieving clinically meaningful outcomes in digital health: a six-step, cyclical precision engagement framework (ENGAGE). Front Digit Health.

[94] Longhini J, Canzan F, Zambiasi P, Toccoli S, Gios L, Del Greco M, Sforzin S, Moz M, Fracchetti M, Saiani L, Brolis R, Guarnier A, Soverini M, Maines M, Ambrosi E (2023). A nurse-led model of care with 2 telemonitoring to manage patients with heart failure in primary health care: a mixed-method feasibility study [response to letter]. Patient Prefer Adherence.

[95] Turnbull S, Cabral C, Hay A, Lucas PJ (2020). Health equity in the effectiveness of web-based health interventions for the self-care of people with chronic health conditions: systematic review. J Med Internet Res.

[96] Ge H, Li J, Hu H, Feng T, Wu X (2025). Digital exclusion in older adults: a scoping review. Int J Nurs Stud.

[97] Hepburn J, Williams L, McCann L (2025). Barriers to and facilitators of digital health technology adoption among older adults with chronic diseases: updated systematic review. JMIR Aging.

[98] Birati Y, Tzemah-Shahar R (2026). Barriers to digital health adoption in older adults: scoping review informed by innovation resistance theory. J Med Internet Res.

