# Supplementary material for: Effectiveness of Digital Health Interventions to Improve Self-Care in Patients With Chronic Diseases: Systematic Review and Meta-Analysis of Randomized Controlled Trials
Source: J Med Internet Res. 2026 Jun 9;28:e88708. doi: 10.2196/88708 (PMC13291736; doi:10.2196/88708)
Supplement: Multimedia Appendix 2 [file jmir_v28i1e88708_app2.docx]

# Supplementary File 2. Search strings

**PUBMED**

("self care"[title/abstract] OR "self-care "[title/abstract] OR "self-management"[title/abstract] OR "medication adherence"[title/abstract] OR "symptom manag*"[title/abstract] OR "behaviour chang*"[title/abstract] OR hospitaliz*[title/abstract] OR "therapeutic adherence"[title/abstract] OR "drug adherence"[title/abstract] OR "medication compliance"[title/abstract] OR "drug compliance*"[title/abstract] OR "symptom* management"[Title/Abstract] OR "treatment compliance"[Title/Abstract]) AND ("Mobile Applications"[Mesh] OR web-based[title/abstract] OR "Robot*"[title/abstract] OR "Wearable Electronic Devices"[Mesh] OR "technolog*"[Title/abstract] OR "mobile health*"[Title/abstract] OR "mhealth*"[Title/abstract] OR "m-health*"[Title/abstract] OR "telehealth*"[Title/abstract] OR "digital health"[Title/abstract] OR "app"[Title/abstract] OR "smartphone*"[Title/abstract] OR "phone application*"[Title/abstract] OR "telephone application*"[Title/abstract] OR "mobile application*"[Title/abstract] OR "health application*"[Title/abstract] OR "internet*"[Title/abstract] OR "world wide web*"[Title/abstract] OR "ipad*"[Title/abstract] OR "laptop*"[Title/abstract] OR "palmtop*"[Title/abstract] OR "palm top*"[Title/abstract] OR "personal digital assistant*"[Title/abstract] OR "interactive voice response*"[Title/abstract] OR "multimedia"[Title/abstract] OR "Mhapps"[Title/abstract] OR "mobile phone*"[Title/abstract] OR "game*"[Title/abstract] OR "gaming"[Title/abstract] OR "gamification"[Title/abstract] OR "whatsapp*"[Title/abstract] OR "e-coach*"[Title/abstract] OR "wearable*"[Title/abstract] OR "smartwatch"[Title/abstract] OR "social media"[Title/abstract] OR "social network*"[Title/abstract] OR "chatbox*"[Title/abstract] OR "chat*"[Title/abstract] OR "robot*"[Title/abstract] OR tele-consultation[Title/abstract] OR "video consultation*"[Title/abstract] OR "remote monitoring"[Title/abstract] OR "telemonitoring"[Title/abstract] OR "remote visit*"[Title/abstract] OR "remote consultation*"[Title/abstract] OR tele-visit*[Title/abstract] OR "video call*"[Title/abstract] OR telemedicine[Title/Abstract] OR "tele health"[Title/Abstract] OR "tele-health"[Title/Abstract] OR textmessage[Title/Abstract] OR "tele consultation"[Title/Abstract] OR teleconsultation[Title/Abstract] OR "tele treatment"[Title/Abstract] OR teletreatment[Title/Abstract] OR "virtual medicine"[Title/Abstract] OR "on-line"[Title/Abstract] OR online[Title/Abstract] OR computer[Title/Abstract] OR tablet[Title/Abstract] OR "digital tools"[Title/Abstract] OR ChatGPT[Title/Abstract] OR telegram[Title/Abstract] OR "text message"[Title/Abstract] OR "health technolog*"[Title/Abstract] OR software[Title/Abstract] OR "implantable device*"[Title/Abstract] OR "portable device*"[Title/Abstract] OR "switchboard service*"[Title/Abstract] OR call*[Title/Abstract] OR "health information"[Title/Abstract] OR exchange*[Title/Abstract]) AND (experimental[Title/Abstract] OR trial[Title/Abstract] OR RCT[Title/Abstract] OR clinical trials, randomized[MeSH Terms] OR controlled clinical trials, randomized[MeSH Terms] OR clinical trial[MeSH Terms]) NOT (pregnan*[Title] OR gestation*[Title] OR gravidit*[Title] OR prison[Title] OR jail[Title]))

**CINAHL**

(TI self-care OR AB self-care) OR (TI self-manag* OR AB self-manag*) OR (TI "symptom manag*" OR AB "symptom manag*") OR (TI "behaviour chang*" OR AB "behaviour chang*") OR (TI hospitaliz* OR AB hospitaliz*) OR (TI hospitalis* OR AB hospitalis*) OR (TI "therapeutic adherence" OR AB "therapeutic adherence") OR (TI "drug adherence" OR AB "drug adherence") OR (TI "medication compliance" OR AB "medication compliance") OR (TI "drug compliance*" OR AB "drug compliance*") OR (TI "emergency access*" OR AB "emergency access*") OR (TI "emergency use" OR AB "emergency use") OR (TI "health service utilization*" OR AB "health service utilization*") OR (TI "health service use" OR AB "health service use") OR (TI "emergency utilization*" OR AB "emergency utilization*") OR (TI "hospital use" OR AB "hospital use") OR ((TI admission* OR AB admission*)) ((TI "clinical complication*" OR AB "clinical complication*")) OR ((TI "symptom* management" OR AB "symptom* management")) OR ((TI "treatment compliance" OR AB "treatment compliance")) OR ((TI "disease* exacerbation*" OR AB "disease* exacerbation*")) OR ((TI exacerbation OR AB exacerbation)))) AND (((OR (TI web-based OR AB web-based) OR (TI Robot* OR AB Robot*) OR (MH "Wearable Electronic Devices+") OR (TI technolog* OR AB technolog*) OR (TI "mobile health*" OR AB "mobile health*") OR (TI mhealth* OR AB mhealth*) OR (TI m-health* OR AB m-health*) OR (TI telehealth* OR AB telehealth*) OR (TI "digital health" OR AB "digital health") OR (TI app OR AB app) OR (TI smartphone* OR AB smartphone*) OR (TI "phone application*" OR AB "phone application*") OR (TI "telephone application*" OR AB "telephone application*") OR (TI "mobile application*" OR AB "mobile application*") OR (TI "health application*" OR AB "health application*") OR (TI internet* OR AB internet*) OR (TI "world wide web*" OR AB "world wide web*") OR (TI ipad* OR AB ipad*) OR (TI laptop* OR AB laptop*) OR (TI palmtop* OR AB palmtop*) OR (TI "palm top*" OR AB "palm top*") OR (TI "personal digital assistant*" OR AB "personal digital assistant*") OR (TI "interactive voice response*" OR AB "interactive voice response*") OR (TI multimedia OR AB multimedia) OR (TI Mhapps OR AB Mhapps) OR (TI "mobile phone*" OR AB "mobile phone*") OR (TI game* OR AB game*) OR (TI gaming OR AB gaming) OR (TI gamification OR AB gamification) OR (TI whatsapp* OR AB whatsapp*) OR (TI e-coach* OR AB e-coach*) OR (TI wearable* OR AB wearable*) OR (TI smartwatch OR AB smartwatch) OR (TI "social media" OR AB "social media") OR (TI "social network*" OR AB "social network*") OR (TI chatbox* OR AB chatbox*) OR (TI chat* OR AB chat*) OR (TI robot* OR AB robot*) OR (TI tele-consultation OR AB tele-consultation) OR (TI "video consultation*" OR AB "video consultation*") OR (TI "remote monitoring" OR AB "remote monitoring") OR (TI telemonitoring OR AB telemonitoring) OR (TI "remote visit*" OR AB "remote visit*") OR (TI "remote consultation*" OR AB "remote consultation*") OR (TI tele-visit* OR AB tele-visit*) OR (TI "video call*" OR AB "video call*") OR ((TI telemedicine OR AB telemedicine)) OR ((TI "tele health" OR AB "tele health")) OR ((TI tele-health OR AB tele-health)) OR ((TI textmessage OR AB textmessage)) OR ((TI "tele consultation" OR AB "tele consultation")) OR ((TI teleconsultation OR AB teleconsultation)) OR ((TI "tele treatment" OR AB "tele treatment")) OR ((TI teletreatment OR AB teletreatment)) OR ((TI "virtual medicine" OR AB "virtual medicine")) OR ((TI on-line OR AB on-line)) OR ((TI online OR AB online)) OR ((TI computer OR AB computer)) OR ((TI tablet OR AB tablet)) OR ((TI "digital tools" OR AB "digital tools")) OR ((TI ChatGPT OR AB ChatGPT)) OR ((TI telegram OR AB telegram)) OR ((TI "text message" OR AB "text message")) OR ((TI "health teconolog*" OR AB "health teconolog*")) OR ((TI software OR AB software)) OR ((TI "implantable device*" OR AB "implantable device*")) OR ((TI "portable device*" OR AB "portable device*")) OR ((TI "switchboard service*" OR AB "switchboard service*")) OR ((TI call* OR AB call*)) OR ((TI "health information" OR AB "health information")) OR ((TI exchange* OR AB exchange*)))) AND ((((TI experimental OR AB experimental)) OR ((TI trial OR AB trial)) OR ((TI RCT OR AB RCT)) OR ((MH "clinical trials, randomized+")) OR ((MH "controlled clinical trials, randomized+")) OR ((MH "clinical trial+")))) NOT (((TI pregnan* OR AB pregnan*)) OR ((TI gestation* OR AB gestation*)) OR ((TI gravidit* OR AB gravidit*)) OR ((TI prison OR AB prison)) OR ((TI jail OR AB jail))))

**PsycINFO**

OR self-care.ti,ab. OR self-manag*.ti,ab. OR "symptom manag*".ti,ab. OR "behaviour chang*".ti,ab. OR hospitaliz*.ti,ab. OR hospitalis*.ti,ab. OR "therapeutic adherence".ti,ab. OR "drug adherence".ti,ab. OR "medication compliance".ti,ab. OR "drug compliance*".ti,ab. OR "emergency access*".ti,ab. OR "emergency use".ti,ab. OR "health service utilization*".ti,ab. OR "health service use".ti,ab. OR "emergency utilization*".ti,ab. OR "hospital use".ti,ab. OR (admission*.ti,ab.) OR OR ("clinical complication*".ti,ab.) ("treatment compliance".ti,ab.) OR (exacerbation.ti,ab.))) AND / OR web-based.ti,ab. OR Robot*.ti,ab. OR exp "Wearable Electronic Devices"/ OR technolog*.ti,ab. OR "mobile health*".ti,ab. OR mhealth*.ti,ab. OR m-health*.ti,ab. OR telehealth*.ti,ab. OR "digital health".ti,ab. OR app.ti,ab. OR smartphone*.ti,ab. OR "phone application*".ti,ab. OR "telephone application*".ti,ab. OR "mobile application*".ti,ab. OR "health application*".ti,ab. OR internet*.ti,ab. OR "world wide web*".ti,ab. OR ipad*.ti,ab. OR laptop*.ti,ab. OR palmtop*.ti,ab. OR "palm top*".ti,ab. OR "personal digital assistant*".ti,ab. OR "interactive voice response*".ti,ab. OR multimedia.ti,ab. OR Mhapps.ti,ab. OR "mobile phone*".ti,ab. OR game*.ti,ab. OR gaming.ti,ab. OR gamification.ti,ab. OR whatsapp*.ti,ab. OR e-coach*.ti,ab. OR wearable*.ti,ab. OR smartwatch.ti,ab. OR "social media".ti,ab. OR "social network*".ti,ab. OR chatbox*.ti,ab. OR chat*.ti,ab. OR robot*.ti,ab. OR tele-consultation.ti,ab. OR "video consultation*".ti,ab. OR "remote monitoring".ti,ab. OR telemonitoring.ti,ab. OR "remote visit*".ti,ab. OR "remote consultation*".ti,ab. OR tele-visit*.ti,ab. OR "video call*".ti,ab. OR (telemedicine.ti,ab.) OR ("tele health".ti,ab.) OR (tele-health.ti,ab.) OR (textmessage.ti,ab.) OR ("tele consultation".ti,ab.) OR (teleconsultation.ti,ab.) OR ("tele treatment".ti,ab.) OR (teletreatment.ti,ab.) OR ("virtual medicine".ti,ab.) OR (on-line.ti,ab.) OR (online.ti,ab.) OR (computer.ti,ab.) OR (tablet.ti,ab.) OR ("digital tools".ti,ab.) OR (ChatGPT.ti,ab.) OR (telegram.ti,ab.) OR ("text message".ti,ab.) OR ("health teconolog*".ti,ab.) OR (software.ti,ab.) OR ("implantable device*".ti,ab.) OR ("portable device*".ti,ab.) OR ("switchboard service*".ti,ab.) OR (call*.ti,ab.) OR () OR ("health information".ti,ab.) OR (exchange*.ti,ab.))) AND (((experimental.ti,ab.) OR (trial.ti,ab.) OR (RCT.ti,ab.) OR (exp "clinical trials, randomized"/) OR (exp "controlled clinical trials, randomized"/) OR (exp "clinical trial"/))) NOT ((pregnan*.ti,ab.) OR (gestation*.ti,ab.) OR (gravidit*.ti,ab.) OR (prison.ti,ab.) OR (jail.ti,ab.)))

**SCOPUS**

(TITLE-ABS-KEY("self care" OR "self-care" OR "self-management" OR "medication adherence" OR "symptom manag*" OR "behaviour chang*" OR hospitaliz* OR "therapeutic adherence" OR "drug adherence" OR "medication compliance" OR "drug compliance*" OR "symptom* management" OR "treatment compliance")) AND (TITLE("Mobile Applications" OR web-based OR "Robot*" OR "Wearable Electronic Devices" OR technolog* OR "mobile health*" OR mhealth* OR m-health* OR telehealth* OR "digital health" OR app OR smartphone* OR "phone application*" OR "telephone application*" OR "mobile application*" OR "health application*" OR internet* OR "world wide web*" OR ipad* OR laptop* OR palmtop* OR "palm top*" OR "personal digital assistant*" OR "interactive voice response*" OR multimedia OR Mhapps OR "mobile phone*" OR game* OR gaming OR gamification OR whatsapp* OR e-coach* OR wearable* OR smartwatch OR "social media" OR "social network*" OR chatbox* OR chat* OR robot* OR tele-consultation OR "video consultation*" OR "remote monitoring" OR telemonitoring OR "remote visit*" OR "remote consultation*" OR tele-visit* OR "video call*" OR telemedicine OR "tele health" OR "tele-health" OR textmessage OR "tele consultation" OR teleconsultation OR "tele treatment" OR teletreatment OR "virtual medicine" OR on-line OR online OR computer OR tablet OR "digital tools" OR ChatGPT OR telegram OR "text message" OR "health technolog*" OR software OR "implantable device*" OR "portable device*" OR "switchboard service*" OR call* OR "health information" OR exchange*)) AND (TITLE-ABS-KEY(experimental OR trial OR RCT OR "clinical trial" OR "randomized controlled trial" OR "controlled clinical trial")) AND NOT (TITLE(pregnan* OR gestation* OR gravidit* OR prison OR jail))
