# Supplementary material for: Effectiveness of Digital Health Interventions to Improve Self-Care in Patients With Chronic Diseases: Systematic Review and Meta-Analysis of Randomized Controlled Trials
Source: J Med Internet Res. 2026 Jun 9;28:e88708. doi: 10.2196/88708 (PMC13291736; doi:10.2196/88708)
Supplement: Multimedia Appendix 3 [file jmir_v28i1e88708_app3.docx]

# Supplementary File 3. Characteristics of the studies included

| First author's surname, year  Country | Recruiting location | Population | N. patients enrolled and lost on follow-up | Intervention | Control care | Data Collection Tool  Follow-up | Follow-up |
| --- | --- | --- | --- | --- | --- | --- | --- |
| Hoban 2013  USA | No profit home healthcare agencies  Home + digital intervention | Patients a primary diagnosis of HF in the agency data enrolled in the HHA for home care services.  78.4 years (NA) | N=80; Dropout=not reported. | Same as control group + telemonitoring equipment. Data were monitored by a telemonitor nurse coordinator daily or more frequently who contacted the patient, primary nurse, or physician when any changes in or missing data occurred.  The telemonitor nurse coordinator and cardiac nursing team (experienced home healthcare registered nurses with a strong cardiac background) | Home visits 2-3 times/week, teaching on HF medications, low-sodium diet, fluid restrictions, daily weights, physical activity, HF booklet with symptoms listed and diary for weights | Self-Care of Heart Failure Index (Riegel 2004) |  |
| Kirwan 2013  Australia | Database of registered patients;  national newsletter;  online diabetes community forum | Type 1 DM > 6 months; HbA1c > 7.5%, treated with multiple daily injections or insulin pump; 18 - 65 years; owner of a smartphone (iPhone)  Age, mean (SD)  I=35.97 (9.64); C=34.42 (10.26) | N=72; I=36; C=36; Dropout=I=11, C=8 (total 19; 26.4%). | GLUCOSE BUDDY APP: enter blood glucose levels, insulin dosages, other medications, diet (food in grams), and physical activity (minutes); For the first 6 months, at least one personalized text message per week (log feedback, diabetes questions, educational tips, and positive reinforcement). The data was reviewed by a CDE (Certified Diabetes Educator) | Usual care (one visit to the diabetes doctor every 3 months). | SDSCA | 9 months |
| Arora 2014  USA | Hospital | Type 2 DM; Hb A1C >= 8%; speak and read English or Spanish; use text messages on their mobile phones  Age | N=128; I=64; C=64; Dropout=I=17, C=19 (total 36; 28.1%). | The *TExT-MED* (Trial to Examine Text Message-Based mHealth in Emergency Department Patients with Diabetes) program was a unidirectional text-messaging intervention targeting low-income adults with poorly controlled diabetes. Participants received two daily text messages for six months in English or Spanish — one educational or motivational message (covering glucose control, diet, exercise, and blood pressure) and one reminder or challenge message encouraging daily health behaviors. Additional weekly messages included medication reminders, healthy living challenges, and trivia quizzes based on the National Diabetes Education Program. Messages were written at a 5th-grade reading level and culturally tailored for Latino patients.  Physicians and diabetes educators provided oversight and reviewed adherence outcomes. | Usual care | - Morisky Medication Adherence Scale - SDSCA | 6 months |
| Heisler  2014  USA | Database | Type 2 DM; HbA1c>7.5% in the previous 6 months; concerns expressed about current diabetes medications during screening assessment  Age, mean (SD)  I = 51 (8,6); C= 52 (9.4) | N=188; I=93; C=95; Dropout=I=6, C=6 (total 12; 6.4%). | One-on-one, face-to-face session (~2 h) with a Community Health Worker (CHW) using the iDecide/iDecido interactive decision aid on a tablet (3G). CHW role: Reviewed tailored content; elicited questions; identified barriers to medication use; helped develop personalized action plan; supported preparation of questions for the next medical visit; scheduled follow-up clinic visit if needed.  CHW phone calls at 3 and 6 weeks to review questions, clinic visit outcomes, and progress on goals/action steps. Tool content: Visual, low–literacy–friendly adaptation of AHRQ guides; Four sections: Animations on diabetes pathophysiology & effects of medication/food/activity, Tailored risk graphs for complications (based on HbA1c), Medication & barriers review with interactive "issue cards" (cost, side effects, weight, dosing), Goal setting & action planning for barriers or concerns. Deep personalization with baseline data; motivational interviewing prompts to support autonomy and change. | One-on-one, face-to-face session (~1.5 h) with a CHW using standard printed AHRQ guides ("Pills for Type 2 Diabetes," "Premixed Insulin for Type 2 Diabetes"). CHW role: Reviewed printed content; elicited questions; identified barriers; helped create an action plan and a list of questions for the provider; scheduled follow-up clinic visit if needed. CHW phone calls at 3 and 6 weeks (same as intervention). Material content: Information on type 2 diabetes and medication classes (oral & insulin) with HbA1c effect, administration, cost, side effects, complication risk. Suggested provider questions, notetaking prompts, tables/graphs, patient photos. | Morisky Medication Adherence Scale | 3 months |
| Boyne  2014  The Netherlands | Hospital | ≥18 years old; Heart failure; an episode of fluid retention requiring diuretics; left ventricular ejection fraction ≤40%, or preserved ejection fraction with diastolic dysfunction.  Age | N=382; I=197; C=185; Dropout=I=38, C=43 (total 81; 21.2%). | Telemonitoring device (Health Buddy®); Daily preset dialogues and questions about symptoms, disease knowledge, and health behavior: patients answer by pressing one of the four keys; responses are sent via a secure server to nurses' computers, and responses are automatically classified into risk profiles: low, medium, or high risk. Positive symptom answers trigger a high-risk alert and prompt immediate action by the heart failure (HF) nurse. If patients answer incorrectly to knowledge or behavior questions (even if not high risk), the device shows the correct answer on screen to educate and improve self-care. A HF nurse and a nurse assistant run the program: The nurse assistant manages educational needs and general high-risk alerts (e.g., Dropoutstent nonadherence, signs of depression). The HF nurse handles urgent high-risk alerts related to symptoms. If signs of depression appear, the patient is contacted and evaluated for psychological support. | Oral and written information, easy access to the heart failure nurse, and four scheduled office visits during follow-up. | - EHFSCB 12 items - Heart Failure Compliance Scale | 12 months |
| Jahangard  Rafsanjani  2015  Iran | pharmacy | Type 2 DM; patients' ability to use a self-monitoring blood glucose device;  Age, mean (SD)  I = 57.3 (8.6)  C = 55.9 (8.7) | N=101; I=51; C=50; Dropout=I=6, C=10 (total 16; 15.8%). | 5 follow-up visits (30 minutes, once a month) with the pharmacist, with a phone call between visits to strengthen adherence to treatment. Blood glucose self-monitoring device, documentation of blood glucose levels every other day (fasting, postprandial, before lunch, before bed), and diabetes medication information leaflets. The checks concerned problems with drugs, self-medication, and the diary.  Patients were referred to the doctor to change their therapy or if the disease was not under control. | The community pharmacist provided a brief education on diabetes self-care and helped them find an appropriate diabetes education program. | Morisky Medication Adherence Scale | 5 months |
| Vuorinen  2014  Finland | Outpatient cardiology | 1) diagnosis of systolic heart failure, (2) age of 18-90 years, (3) NYHA class ≥2 (an interview-based classification by the New York Heart Association concerning limitations to physical activity), (4) left ventricular ejection fraction ≤35% as measured during hospital visits, (5) need for a regular check-up visit, and (6) time from the last visit of less than 6 months  Age, mean (SD)  I = 58.3 (11.6), C = 57.9 (11.9) | N=94; I=47; C=47; Dropout=I=1, C=0 (total 1; 1.1%). | APP: report symptoms and parameters to the nurse and received feedback; Use of a scale, sphygmomanometer, and instructions for self-medication (cardiology team could access the data); the nurse may invite the patient for a follow-up visit if still necessary after the phone call. If a patient did not comply with the weekly reporting plan, the nurse would contact the patient and encourage them to continue with monitoring. | The cardiology team: monitoring and interpretation of symptoms, optimization of drug therapy, and education. Patients are encouraged to regularly measure their blood pressure, heart rate, and weight at home. Visits to the clinic and by phone. The heart failure nurse contacted patients by phone to motivate them and remind them to comply with the self-medication plan | EHFSCB 12 items | 6 months |
| Park  2014  USA | Community Hospital | ≥ 21 years; hospitalized for non-ST elevation myocardial infarction, ST elevation myocardial infarction, or percutaneous coronary intervention; prescribed an antiplatelet drug; prescribed a statin-based drug; owned a mobile phone with text messaging capabilities; able to speak, read, and understand English.  Age, mean (SD)  I1=58.2 (10.6); I2=58.3 (8.5); C=61.1 (9.1) | N=90; I1=30; I2=30; C=30; Dropout=I1=2, I2=2, C=2 (total 6; 6.7%). | Text messaging system (CareSpeak Health Manager® platform); personalized, automated messages were sent to participants over 30 days. Patients in the “Reminders + Education” group received 74 text messages, including daily medication reminders and educational content on CHD self-care, risk factors, and lifestyle behaviors. Messages were two-way, requiring participants to reply to confirm medication intake. Responses were transmitted to a secure server and automatically logged for adherence tracking. Educational messages reinforced knowledge, motivation, and self-efficacy regarding medication and cardiovascular risk reduction. Nurses monitored responses, followed up on nonresponses, and provided technical or motivational support when needed. | Usual care | Morisky Medication Adherence Scale | 1 month |
| Hägglund  2015  Sweden | 3 teaching hospitals | Heart failure; hospitalized; NYHA class IIIV; treatment with diuretics or prescribed if necessary; referred directly to primary care  Age, mean (SD)  I = 75 (8), C = 76 (7) | N=178; I=90; C=50; Dropout=I=6, C=10 (total 16; 9.0%). | Information sheet, home intervention system (HIS) installed. Self-care tips in advance (weight gain of 2 kg in 3 days, contact the HF center by phone); the tablet was divided into 4 different selectable views; 1) what the patient needed to know nowadays (actual daily weight, dose of the drug, and a brief briefing), 2) overview of HF disease and lifestyle information (exercise, smoking, fluid restriction, vaccination, etc.), 3) graphical representation of changes in weight, medication and wellbeing over time, 4) contact details of the nurses and doctors in charge of the HF center and the people responsible for technical support | Information sheet as the GI with advice on HF treatment and a priority number to call in case of need | EHFSCB 9 items | 3 months |
| Pfaeffli Dale  2015  Auckland, New Zealand | hospitals | Coronary heart disease (myocardical infarction, angina, revascularization); english speakers; Internet access  I(n=61)  Age: 59.0 (10.5),  C (n=62)  Age 59.9 (11.8), | N=123; I=60; C=62; Dropout=I=4, C=3 (total 7; 5.7%). | TEXT4HEART: standard cardiac rehabilitation (CR), care + basic skills provided via text messages + access to a support website + pedometer to self-monitor their physical activity | Usual care: inpatient rehabilitation and encouragement of participation Center-based CR, lifestyle change education, and psychosocial support | Morisky Medication Adherence Scale | 6 months |
| Kamal  2015  Pakistan | hospitals | ≥ 18 years; >1 month since the last stroke episode; at least two medications for control risk factors for stroke; modified ranking score of 3 or lower; personal mobile phone with access at all times (or assistant who has a mobile phone available); Ability to receive, understand and respond to an SMS (or assistants available who can carry out the activities mentioned above)  I = (n = 100)  Age: mean 56.07 (1.5 SD), Males: 64  C= (n=100)  Age: mean 57.62 (1.3 SD), Males: 71 | N=200; I=100; C=100; Dropout=I=17, C=21 (total 38; 19.0%). | The *SMS4Stroke* program provided personalized, prescription-tailored SMS reminders to adult stroke survivors taking multiple medications. Messages were sent daily for two months and included (a) individualized reminders for each prescribed medication and (b) twice-weekly health education messages covering stroke prevention, risk factors, and healthy behaviors. Each SMS was designed using the Health Belief Model and Social Cognitive Theory, customized for the patient’s language (English or Urdu), and timed to coincide with medication schedules. Participants were required to confirm receipt by replying to the SMS. Follow-up SMS reminders and feedback were automated using FrontlineSMS software.A team of neurologists, behavioral scientists, and trained nurses developed the message library and oversaw implementation. Nurses enrolled participants, trained them (or caregivers) in SMS use, monitored responses, and managed follow-ups, while neurologists supervised data analysis and clinical outcomes. | Usual care: regular follow-up visits with their neurologist, specialized in stroke. | Morisky Medication Adherence Scale | 2 months |
| Jeon  2016  Korea | Outpatients | Chronic hepatitis B surface antigen test results during the previous 6 months; no comorbidities with CHB (cirrhosis, hepatocellular carcinoma or hepatic failure); 19 – 60 years; using an Android Smartphone  Age  I = 39.4 (10.2); C= 44.7 (9.6) | N=156; I=71; C=85; Dropout=I=4, C=5 (total 9; 5.8%). | Smartphone-based self-care application, inclduing eight interactive modules: *Self-Care* (daily questions on medication, diet, alcohol use, exercise, and follow-up), *Disease Knowledge* (educational content on anatomy, symptoms, infection, treatment, and vaccination), *Statistics* (graphs of health trends), *Liver Lab Record* (tracking of GOT/GPT results), *My Information*, *Alarms* (reminders for medication, clinic visits, and self-care), *Role Practices* (peer support board), and *App Information*. Patients used the app for 12 weeks, receiving weekly group push messages from the research team to encourage engagement and reinforce adherence. A research assistant acted as *app manager* via a separate control interface, monitoring activity, managing discussion boards, and sending motivational messages. Nurse researchers trained participants on app installation and use, provided educational and technical support, and monitored self-care performance data, while a gastroenterologist from the partner university hospital oversaw the clinical accuracy of the educational content and patient eligibility. | Usual care | SelfCare Performance Tool | 3 months |
| AkhuZaheya  2016  Northern Jordan | hospitals | Cardiovascular diseases at least three months ago (hypertension or heart disease); ≥ 18 years; having an active phone number and a mobile phone, which can receive text messages via mobile phone  Age: mean (SD)  54.94 (10.857) | N=180; I=60; P=60; C=60; Dropout=I=8, P=8, C=4 (total 20; 11.1%). | An SMS-based tele-nursing intervention sending personalized text reminders to encourage adherence to prescribed medication, healthy diet, and smoking cessation among cardiovascular disease patients. Messages were delivered daily for three months and framed according to Self-Regulation Theory, emphasizing motivation, responsibility, and self-care reinforcement.  The program was organized by nurses. | Control group who received usual care, including scheduled medical visits to cardiology clinics, the performance of certain diagnostic procedures and laboratory tests and the prescription of the drugs usually taken.  Placebo group, which received general messages daily, including health advice in addition to usual care, same frequency as intervention. | Morisky Medication Adherence Scale | 3 months |
| Kim  2016  USA | Study participants were employees and family members insured by Scripps Hpark  ealth | Hypertension/diabetes/ cardiac arrhythmia, Being able to participate in visits to a Scripps facility; access the Internet; participating in the HealthComp disease management program; willing to use wireless devices, iPhones, and the HealthyCircles platform.  Age  I = 57.5 (SD 8.6), C= 57.7 (SD 8.7) | N=160; I=75; C=85; Dropout=30 (18.8%). | A wireless self-monitoring program integrating a Bluetooth-enabled blood pressure monitor, mobile app, and web-based portal for disease management. Participants recorded blood pressure readings, received automated reminders to measure daily, and could review color-coded feedback and educational tips through the app. The program also included a secure website where participants tracked lifestyle behaviors (exercise, diet, alcohol, and smoking) and reviewed trends with health tips tailored to their data. The intervention lasted six months and was designed to strengthen patient activation and adherence to hypertension management behaviors.  Participants were trained by nurses and clinical researchers, who reviewed data remotely, followed up on abnormal readings, and collaborated with supervising physicians to adjust care plans. | Standard disease management  program | Morisky Medication Adherence Scale | 6 months |
| Koufopoulos  2016  UK | university | Asthma; has not taken part in previous studies; be prescribed an ICS prevention/control inhaler for a weekly regimen of at least one dose per week | N=216; I=99; C=117; Dropout=I=60, C=53 (total 113; 52.3%). | A 9-week online community and mobile platform for adults with asthma prescribed inhaled corticosteroids. Participants logged each medication use and could read and reply to others’ posts, sharing experiences about adherence and self-management. The platform aimed to enhance social support, accountability, and motivation to maintain treatment adherence. Managed by the research team. The online system was automated, with researchers overseeing site operation and data collection; no direct health professional input or individualized feedback was provided. | An online diary (Asthma Diary) created using Google Forms. A single-item survey has been created: "How many times  Did you get your quote?" Participants could then enter the number of puffs | SMAQ Scores | 9 weeks |
| Baron  2017  UK | hospital | ≥ 18 years; poorly controlled type 1 or type 2 diabetes (most recent HbA1c to be hired in the last 12 months and over 7.5%); knowledge of the English language.  Age: mean 57.2 (13.6 SD) | N=81; I=45; C=36; Dropout=I=6, C=5 (total 11; 13.6%). | Mobile-Phone–Based Home Telehealth (MTH) system; participants used a smartphone to record and transmit blood glucose (BG), blood pressure (BP), insulin dose, weight, physical activity, and meal timing to the telehealth platform. The system provided automated, color-coded graphical feedback on BG and BP (blue: hypoglycemia, green: normal, amber: borderline, red: hyperglycemia) visible on the phone display. Out-of-range readings triggered nurse-initiated feedback. Participants also received six-weekly educational phone calls on diabetes management and motivation.  A diabetes specialist nurse monitored transmitted data during working hours, contacted patients when readings were abnormal, and provided education, behavioral reinforcement, and medication titration guidance in collaboration with the clinical diabetes team. | Usual care | SDSCA | 9 months |
| Melin  2018  Sweden | University hospitals | aged ≥ 18 years who are hospitalized for heart failure during their hospital stay or hospital follow-up visit within 4 weeks of surgery unloading  I (n= 32)  Age: 75 (8)  C (n=40)  Age: 76 (7) | N=82; I=32; C=40; Dropout=I=5, C=17 (total 22; 26.8%). | OPTILOGG: tablet computer locked to custom software and wirelessly connected to a scale. The patient is asked to step on the scale every morning.  The weight is transferred wirelessly to the tablet, and the current day's dose of diuretics is displayed on the screen. The weight level for each patient is defined by the specialist and the nurse. The patient is also given a brief suggestion on how to improve life with heart failure. The educational module includes information on the importance of taking prescribed medications, lifestyle changes, recipes for healthy foods, facts about HF, and symptoms. Every 5 days, patients enter their symptoms (fatigue, shortness of breath, and swelling/edema). | Usual caremars | EHFScBS 9 items | 6 months |
| Desteghe  2018  Belgium | house | Atrial Fibrillation patients who are already taking or starting therapy once daily (OD) NOAC rivaroxaban or NOAC apixaban twice daily (BID)  Patients treated with NOAC BID (n=24)  Age: 73.1 (8.5),  Patients receiving NOAC OD therapy (n = 24)  Age: 70.2 (8.5), | N=48; I=24; C=24; Dropout=1 (2.1%). | Using the Electronic Medication Assessment System  Event monitoring system (MEMS): a special cap that fits a medicine bottle by recording the exact date and time of opening the bottle. In the first two telemonitoring phases, patients had to place the drug bottle with the MEMS cap on a wireless reader after each drug intake. Next, the information from the hood was automatically and wirelessly transmitted to an internet server. For the last observation phase, patients had to hand over the wireless reader and use only their MEMS drug bottle, which continued to record daily intake but without the known telemonitoring transmissions. During the feedback phase, patients received a phone call. MedAmigo software was used to review daily data adherence on weekdays: an online assessment to give feedback directly to the patient during the "feedback phase". | The study design included three periods of 3 months each, with a crossover between the first two study periods:  Tm, telemonitoring phase;  Tm + F, telemonitoring  with an additional feedback phase | Morisky Medication Adherence Scale | 9 months |
| Kamal  2018  Pakistan | hospital | ≥ 18 years; history of CVA or CAD; ≥ 1 month since the last episode of CVA or CAD; Use of antiplatelet agents and statins; mobile phone with access at all times (or always had a main assistant who had a mobile phone); Ability to receive and understand an SMS in English or Urdu (or always have an assistant available who could carry out the activities mentioned above homework)  I (n=99)  Age: 59.1 (11.6),  C (n=98)  Age in years: 57.7 (11.1), | N=201; I=99; C=98; Dropout=I=13, C=6 (total 19; 9.5%). | The *Talking Rx* intervention used a tailored health information technology platform designed to improve medication adherence among stroke and heart attack survivors. It combined daily Interactive Voice Response (IVR) calls, which delivered personalized, pre-recorded audio messages reminding participants to take their prescribed statins and antiplatelets, with weekly SMS messages focused on lifestyle modification, including diet, exercise, and smoking cessation. Calls and messages were available in Urdu and tailored to literacy level; the system logged patient responses to verify engagement and adherence. Each participant received the program for three months. The program was delivered by a multidisciplinary team, including neurologists, cardiologists, and nurse coordinators. Nurses trained participants in using the IVR system, ensured phone access, and reviewed adherence data generated by the digital platform. Clinical oversight and message development were led by physicians from the Aga Khan eHealth Resource Centre and Stroke Service. | Usual care: regular follow-up visits with the neurologist or cardiologist specializing in stroke | Morisky Medication Adherence Scale | 3 months |
| Morawski 2018  USA | Rural and urban places | 18 - 75 years; systolic blood pressure equal to or greater than 140 mmHg on treatment  with at least 1, but not more than 3 first-line antihypertensive drugs  I (n= 209)  Age: 51.7 (10.5),  C (n= 202)  Age: 52.4 (10.1), | N=412; I=210; C=202; Dropout=I=23, C=23 (total 46; 11.2%). | Medisafe APP: to help individuals adhere to prescribed therapies. First, drug lists are entered manually, along with the preferred time of administration, or are automatically populated via a link to an existing medical record. When this integration was established, the app provided alerts to patients when it's time to take medication and generated weekly reports on medication adherence. Monitoring of blood pressure and other biometric measurements. Users can designate a "Medfriend" who is granted access to the patient's medication intake history, who receives alerts when doses are missed, and who can provide peer support | Usual care | Morisky Medication Adherence Scale | 3 months |
| Schnall 2018  USA | HIV Clinic and CommunityBased Organizations | ≥ 18 years; HIV; experienced at least 2 of 13 HIV-related symptoms in the past week;  Mini-Mental State Examination (MMSE) ≥ 24/30; smartphone or tablet; speaks English  I (n=40)  Age: 50.0 (11.7),  C (n = 40)  Age: 50.8 (9.0), | N=80; I=40; C=40; Dropout=I=3, C=1 (total 4; 5.0%). | Upon enrollment, participants installed a web app shortcut to access mVIP, select an avatar, and log in weekly to assess symptoms and receive personalized self-care strategies. Both groups used the mVIP app, but only the intervention group received tailored strategies and short animated videos (3–27 seconds) illustrating them. Each week, participants reported the presence and severity of 13 symptoms, after which the app suggested three self-care strategies to try. The app included a reminder system that emailed participants at 7:30 p.m. on days 7, 14, 18, and 21 after their last use to encourage continued engagement. | Mobile app without self-care strategies | CASE Adherence Index |  |
| Agarwal  2019  Canada | hospital | ≥ 18 years; Type 2 DM; HbA1c ≥8.0% in the last 3 months; email active or able and willing to obtain one; Read English language  I= 110  Age= 51.5(10.6),  C=113  Age=52.1 (10.7), | N=223; I=110; C=113; Dropout=I=38, C=46 (total 84; 37.7%). | BlueStar APP for 3 months: patients could enter information about their T2DM management into the app (baseline health, daily blood sugar, physical activity, and food intake). The app used this data to deliver real-time, personalized messages to motivate, educate, and influence behavior. The app also facilitated the transfer of data to the doctor through Smart Visit reports. | Usual care | SDSCA | 3 months |
| Sun 2019  China | Hospital | Patients with CHF NYHA class >II  IG: 68.21 (4.69) CG: 68.57 (4.12) | N=100; Dropout=0 (0%). | Personalized health education plan with daily health education content on HF knowledge, treatment plan and goals, medication, exercise, nutrition, and prevention of acute attack over 3 days to help patients correct their self-care plan and monitored its implementation during hospitalization. After discharge: internet-based medical platform with a nurse available 9-17 daily + weekly health education program + self-care plan + messages sent with APP platform or WeChat public account + weekly phone calls for 3 months, 1 time/every 2 weeks after 3-4 months, 1 time/months after 5–6 months. Meeting of research team members every 2 months where patients or families were invited.  Cardiology nurse, nursing staff | Nursing guidance at discharge with a health education manual + phone calls 2 weeks after discharge for monitoring and health guidance | Self-Care of Heart Failure Index (Riegel 2009)  Self-care maintenance and management |  |
| Park  2020  Republic of Korea | Hospital | ≥45 years; COPD; GOLD Phase 1, 2, or 3; smartphone and could send text messages; able to communicate  Age, mean (SD)  67.88 ± 10.49 | N=44; I=23; C=21; Dropout=I=1, C=1 (total 2; 4.5%). | Educational material is installed in the smartphone APP. The exercise expert prescribed personalized exercises. An exercise booklet, video clips of exercises, and a pedometer have been provided. Participants recorded the time and type of exercise and the step count from the pedometer in the smartphone app. Participants recorded symptoms, bronchodilator use, and health care use due to exacerbations in the app. Participants were encouraged to communicate with other participants and the research team via text messages in the smartphone app or via call. | The exercise expert has prescribed personalized exercises for each participant. An exercise booklet was provided. The research team called participants to check their health status once a month for 6 months. | The Inventory of SelfCare Behavior for Alberto's Chronic Obstructive Pulmonary Disease | 6 months |
| Stamenova 2020  China | Clinics | COPD; speak and read English; Willingness to participate in a technology monitoring program  Age, mean (SD)  I1=71.76 (7.28); I2=71.98 (9.52); C=72.78(9.16)  Males  I1=23(56); I2=23(56); C=19(48)  With caregiver  I1=83, I2=76, C=75 | N=122; I1=41; I2=41; C=40; Dropout=I1=5, I2=6, C=7 (total 18; 14.8%). | The Medly smartphone app was a home-based heart failure telemonitoring system integrated with Bluetooth-enabled devices (weight scale, BP cuff). Patients entered or automatically uploaded readings daily, and the app generated automated feedback messages based on an algorithm using individualized thresholds. Messages guided patients on self-care actions (e.g., medication adjustment, symptom monitoring) and triggered alerts to the clinical team when abnormal values were detected. Participants also received educational material and reminders. A dedicated heart failure nurse coordinator monitored data through the clinician dashboard, contacted patients as needed, and liaised with cardiologists to address flagged alerts.  . | Usual care | Partners in Health (PIH) Scale | 6 months |
| Ding  2020  Australia | hospital | ≥ 18 years; CHF with low ejection fraction; able to weigh safely; with a personal primary care physician (GP) or agreeing to use a designated general practitioner with a permanent residence address  Me = 91  Age: 69.5 (12.3), Males: 66 (73%)  C = 93  Age: 70.8 (12.8), Males: 75 (81%) | N=184; I=91; C=93; Dropout=I=24, C=10 (total 34; 18.5%). | The ITECCHF consists of Bluetooth-enabled scale connected to a secure call center system. Patients recorded body weight daily; data were transmitted automatically for remote monitoring. Alerts were generated for noncompliance or abnormal readings, prompting follow-up. Nurse care services supported by the ITEC-CHF program team. Nurses monitored data, contacted patients when needed, and provided education and guidance to improve adherence. | Standard package containing a paper diary and the booklet Living well with chronic heart failure.  Patients were asked to continue to follow the usual care for congestive heart failure, provided by congestive heart failure clinics and general practitioners, and to self-manage congestive heart failure as previously indicated. | Heart Failure Compliance Questionnaire | 6 months |
| Si  2020  China | hospital | 18 – 60 years; epilepsy lasting more than 1 year;  more than three seizures during the 6 months; reside in the study area; be proficient in the use of smartphones | N=380; I=190; C=190; Dropout=I=14, C=39 (total 53; 13.9%). | Smartphone app integrated in WeChat®, including a medication calendar, online educational forums and blogs, tools for seizure reporting, video or message consultations, and self-management questionnaires.  Patients used the app daily to record seizures, review educational articles, and receive reminders supporting adherence and lifestyle management.  The program was run by neurologists and epilepsy nurses at the Sichuan Provincial People’s Hospital.  Staff trained participants in app use, monitored online data, confirmed seizure reports, and provided remote guidance and feedback through the WeChat platform. | Usual care | Chinese Epilepsy SelfManagement Scale (CESMS) Score | 6 months |
| Wonggom 2020  Australia | HF outpatient clinic in hospital  Hospital ward/HF clinic + m-health at home | Patients with a diagnosis of HF, NYHA class I to IV | N=36; Dropout=1 (2.8%). | 1 session to teach how to use the app during hospitalization + avatar app on tablet on the topic: understanding HF, looking after yourself, things to do every day, emergency action plan.  Research nurse conducted the intervention. | Bedside education, follow up at the HF clinic, booklet | Self-Care of Heart Failure Index (Riegel 2009) |  |
| Dincer  2020  Turkey | hospital | ≥18 years; Diabetes at least 6 months earlier; no diabetic foot injury; Turkish-speaking individuals; able to use a smartphone; no communication or mental health problems  I = 65  Age: 49.5 (17.4), Males: 34 (52.3)  C = 65  Age: 54.7 (13.6), Males: 32 (49.2) | N=130; I=65; C=65; Dropout=0 (0%). | A mobile diabetic foot care education app (M-DFCE) that delivered interactive animation-based modules covering daily foot inspection, hygiene, footwear selection, and ulcer prevention. The app sent daily push notifications reminding participants to inspect and clean their feet, watch educational animations, and complete short self-assessment quizzes. It also included a digital diary for logging foot-check results and symptoms, allowing participants to track progress over time. The program lasted three months. Nurses provided initial in-person training, monitored digital engagement, and offered individualized advice through phone or online contact for participants reporting abnormalities or nonadherence. | Diabetic foot care training in line with clinical guidelines at the hospital's diabetes teaching room.  The instruction included the provision of printed visual teaching materials detailing daily activities. Patients received education individually via direct instructions, Q&A methods, and discussion | Behavioral Foot SelfCare Scale (FSCBS) | 1 month |
| Hong  2021  Taiwan | Medical Center | >20 years; myocardial infarction; landline; Language proficiency in Mandarin or Taiwanese Chinese  Age, mean (SD)  I=72.8 (9.2), C=71.8 (7.3)  Males  I=71.7%, C=71.7% | N=60; I=30; C=30; Dropout=0 (0%). | Health Information Technology (Health-IT) teleweb system based on Self-Efficacy Theory; installed through the patient’s home landline and connected to a cloud-based data platform. Patients used the system daily for 3 months to upload blood pressure, physical activity, and self-management records. The program provided real-time feedback, automated educational prompts, and interactive nurse counseling sessions focusing on CAD self-management, exercise, diet, and medication adherence. Patients in the waitlist control group received usual care for 3 months before crossing over to receive the same intervention. A team of nurses trained in theory-based self-management education monitored uploaded data, contacted patients weekly, and provided tailored motivational coaching and remote counseling through the Health-IT platform. | Usual care for 3 months and for the next 3 months, the same intervention as the intervention group | Partners in Health (PIH) Scale | 6 months |
| Bruggmann  2021  Switzerland | Hospital | >18 years; myocardial infarction treated with percutaneous coronary intervention; tablet, smartphone or computer; French language skills  Age: medium  I=56, C=62  Males  I= 82%, C=89% | N=68; I=38; C=30; Dropout=I=5, C=3 (total 8; 11.8%). | A smartphone health diary app was developed to support patients with multiple chronic conditions (heart failure, COPD, diabetes) in monitoring their daily health status. The app enabled users to record vital signs (BP, HR, weight), symptoms, medication intake, and physical activity, providing visual trend graphs and alerts when values deviated from set thresholds. It also featured automated feedback messages, reminders for medication and exercise, and a secure data transfer function for clinicians. The intervention was applied for 12 weeks alongside routine care. Specialized nurses introduced patients to the app, provided technical assistance, and followed up via phone to review data entries and adherence. Physicians supervised the clinical review of transmitted data and handled alerts requiring medical action. | Usual care | Adherence to Refills and Medication Scale (ARMS) | 6 months |
| Jiang 2021  Singapore | Hospital  Home + digital intervention | Patients with HF using a smartphone everyday  CG: 68.82 (13.14); IG A: 69.08 (10.51); IG B: 66.82 (11.81) | N=213 randomized; baseline N=177; Dropout: 3 months from baseline 4.5%, 6 months from baseline 8.5%, 6 months from randomization 23.9%. | Usual care + group A and group B.  Both group A and B received the HOM-HEMP intervention as HF self-management program adopting a psychosocial education approach with HF self-management toolkit + 3 home visits (40 min -1 h; 1 every 2 weeks). Educational materials on HF, salty foods, + drinking mugs with marks, scale, pill box with alarms + Educational plan + Motivational interviewing. Group B received a smartphone app with reminders for medication and appointments, weight/blood pressure/ symptom logs, educational information, chat room with nurse  Research nurse trained in MI with 10 years of clinical experience | Medical, nursing, allied health and follow-up services at the hospital | Self-care of heart failure index (Riegel 2009) |  |
| Hsieh  2021  Taiwan | Surgeries | ≥ 20 years; Atrial fibrillation; Ability to use a computer and browse the Internet  Age: Medium (SD)  73.08 (1.,71)  Males: n (%)  116 (50.2) | N=231; I=115; C=116; Dropout=I=1, C=0 (total 1; 0.4%). | A web-based integrated management program developed to promote self-care and medication adherence among patients with atrial fibrillation. Participants accessed the online platform through a computer or tablet to complete interactive educational modules, report symptoms and medication intake, and receive individualized feedback. The system provided alerts for irregular responses, automated reminders for medication schedules, and educational materials on disease management and lifestyle modification. The program lasted 6 months, with data collected over 2 years for long-term outcomes. The intervention was implemented by a multidisciplinary team, including cardiologists, nurses, and IT specialists. Nurses acted as primary contacts, monitoring patient entries, providing feedback via the platform, and offering online support. Cardiologists supervised treatment and collaborated with nurses to interpret monitoring data and adjust therapies as needed. | Usual care, paper manual on the management of AF | MARS | 6 months |
| Ni  2022  China | Flyers and healthcare professionals | ≥18 years; coronary artery disease; antihypertensive medication for at least 90 days after enrollment; Ability to read mobile messages; mobile phone capable of receiving messages via WeChat and reminders from Message Express; Availability of an electronic blood pressure and HR monitor  Age: Medium (SD)  I=61(11); C=62 (11)  Males: n (%)  I=83 (80.6%); C=74 (79.6%) | N=196; I=103; C=93; Dropout=0 (0%). | A dual mobile-application mHealth system integrating WeChat® and Message Express® platforms to support patients with coronary heart disease. Participants in the intervention group received educational messages on cardiovascular health via WeChat and automated medication reminders via Message Express for 60 days, followed by a 30-day follow-up. Messages covered topics such as adherence, symptom management, healthy lifestyle, and prevention of complications. A trained nurse coordinator oversaw message scheduling, participant contact, and monitoring of app use and response rates. | Usual care | Voils Extent | 90 days |
| Ware 2022  Canada | Heart Function Clinic in hospital  Digital intervention | Patients with diagnosed with HF with reduced ejection fraction (<40%), uncontrolled HT (≥140/90 mm Hg auscultatory), or insulin-requiring DM and performing self-capillary glucose monitoring  59 (12.6) for overall sample not only those with HF | N=66; Dropout at 6 months for HF: not available. | Smartphone + Bluetooth devices (weight scale, blood pressure monitor, and blood glucose monitor); instruction to monitor their daily weight, blood pressure, heart rate, and symptoms, and Medly smartphone app. The app was for (a) telemonitoring to record physiological measurements with wireless home medical devices, (b) receiving automated answer symptom questions and self-care instructions based on algorithms (the app send alerts to the clinical team via email). Historical trends were viewable on a secure web portal.  None or nurse practitioner | Clinical visits every 3 to 6 months, optimization of medical therapy, self-management education | Self-Care of Heart Failure Index (Riegel 2009) |  |
| Han  2023  China | Surgeries | DM2 from 5-10 years with HbA1c between 7% and 11%; 18- 75 years; no acute infections, malignancies, liver failure, pregnancy, breastfeeding, prolonged cortisone treatment, or inability to use smartphone applications  Mean age (SD)  I=52.1(9.2); C=51.8(8.3)  Males N (%) | N=418; I=212; C=206; Dropout=I=97, C=141 (total 238; 56.9%). | Telemedicine-assisted structured self-monitoring of blood glucose (SMBG) using a Bluetooth-connected glucose meter (SINOMEDISITE®) linked to the Huayi Glucose Butler App. The device automatically transmitted real-time glucose readings to the app, which displayed color-coded feedback (green: normal, yellow: high, red: low).  The app provided educational material on diabetes, diet, and physical activity, personalized feedback, and secure messaging between patients and physicians.  Weekly phone coaching sessions reinforced motivation, problem solving, and adherence to the SMBG regimen. The program was coordinated by endocrinologists and diabetes educators at the Qingpu Branch of Zhongshan Hospital. They reviewed transmitted data via the telemedicine platform, adjusted treatment plans as needed, and delivered personalized guidance and behavioral coaching. | Usual care | Diabetes Self-Management Questionnaire (DSMQ) | 6 months |
| Poorcheraghi  2023  Iran | hospital | ≥60 years; cardiovascular disease, diabetes, hypertension or COPD; > 5 drugs/day; smartphones; Ability to read and write;  Mean age (SD)  I=69 (5.6); C=68.9 (5.2)  Males: N (%) | N=182; I=92; C=92; Dropout=I=4, C=4 (total 8; 4.4%). | A mobile drug management application was developed specifically for older adults taking multiple medications. The app included personalized medication lists, audio and visual reminders for timing and dosage, and a logging system that tracked adherence and alerted users when doses were missed. It also provided information on each prescribed drug, including side effects and instructions, to reduce confusion and prevent medication errors. Participants used the app daily for 8 weeks to record medication intake, review reminders, and receive real-time prompts to ensure adherence. Nurses verified medication schedules, and provided continuous remote follow-up. They also monitored for nonadherence, medication errors, and potential adverse events, contacting patients when intervention was needed or re-hospitalization risk increased. | Usual care | Morisky Medication Adherence Scale | 10 weeks |
| Deckwart 2023  Germany | Hospitals and cardiology centers | ≥18 years; NYHA II or III; Hospitalization for heart failure (CI) within the past 12 months; Optimized drug treatment; Left ventricular systolic function (LVEF) ≤45%  Mean age (SD)  I=70.3 (10.5); C=70.3 (10.5)  Males: N (%) | N=1571; I=796; C=775; Dropout=I=129, C=121 (total 250; 15.9%). | Remote Patient Management (RPM) system integrated a home telemonitoring kit that automatically transmitted patients’ daily data—ECG, blood pressure, body weight, and oxygen saturation—to a central telemedical service center. The data were analyzed using preset thresholds that triggered alerts for abnormal values or missing information. Patients also received structured education to promote medication adherence, symptom recognition, and self-care behaviors to prevent decompensation.  Nurses reviewed data daily, followed up with patients by phone when alerts occurred, and escalated clinical issues to physicians. Cardiologists supervised care adjustments and collaborated with patients’ local healthcare providers to ensure continuity of treatment and rapid response to early signs of deterioration. | Usual care | EHFScBS 9 items | 12 months |
| Guo 2023  China | Hospital | Adults (18–75 yrs) with type 2 diabetes mellitus, alert, cognitively intact, able to use mobile app  Mean age (SD) I = 55.3 (13.8); C = 59.5 (14.6)  Males: N (%) I = 23 (71.9%); C = 16 (50.0%) | N=68; I=32; C=32; Dropout=4 (5.9%). | mHealth management using a model that integrated a network platform, implantable glucose sensor, mobile app, and GP support. A glucose sensor was implanted subcutaneously for continuous 14-day monitoring, replaced midway through the 4-week program. Blood glucose data were transmitted to a processor that generated dynamic trend graphs and analyzed fluctuations in relation to lifestyle factors. Patients used the app to record health information, view glucose data, and communicate with their GP, while GPs monitored data and provided personalized diet, exercise, and education programs. Interventions were adjusted daily based on real-time glucose trends, with weekly 55–65-minute health education sessions via video or voice call. | Usual care: weekly telephone follow-ups by GPs to record blood glucose values and remind patients to attend clinic reviews. During follow-up visits, patients were provided with educational materials covering four themes: basic knowledge of T2DM, dietary guidance, exercise therapy, and prevention of complications. | SDSCA |  |
| Bernal-Jiménez  2024  Spain | hospital | 18 - 75 years; Have smartphones with internet connection throughout the study period;  have undergone percutaneous coronary intervention (PCI) with stent implantation  Mean age (SD)  I=57.70 (8.16); C=61.46 (9.47)  Males n(%)  I=53 (79%); C=39 (64%); | N=134; I=67; C=67; Dropout=I=0, C=6 (total 6; 4.5%). | mHealth: education on healthy lifestyles, physical activity monitoring, diet, blood pressure, blood sugar, treatment adherence, and smoking cessation.  The app uses the stages of the theory of change: attention, retention, memory, action, and motivation. Capture attention with warnings and bright colors; retention through reminders, repetitions, and graphs; action through instructions, advice, and feedback; and motivation with internal discussions, goal setting, self-monitoring, and feedback. | Usual care | MGL MAQ: Morisky Green and Levine Medication Adherence Questionnaire | 9 months |
| FarzanehRad  2024  Iran | hospital | ≥18 years old; NYHA II or III heart failure;  manage drugs independently; Knowledge of the Farsi language; mobile phone  Median Age (SD)  I=61.7(13); I2=64.2(8.9); C=59.8(10.5)  Males %  I=68.3%; I2=65.1%; C=71.4% | N=189; I1=63; I2=63; C=63; Dropout=I1=13, I2=9, C=8 (total 30; 15.9%). | This study compared two low-cost self-management interventions for patients with chronic heart failure. The Tailored Text Messaging (TTM) group received daily personalized SMS reminders synchronized to each participant’s medication schedule. Messages included motivational phrases, dosing times, and symptom-check prompts. They also provided brief educational advice about fluid restriction, diet, and warning signs of worsening heart failure. The Pillbox group used labeled pill organizers with scheduled compartments and received standard discharge instructions. Both interventions lasted 12 weeks, and adherence was tracked through pill counts and questionnaires (MARS scale).  Nurse educators managed participant onboarding, message scheduling, and phone follow-ups to confirm understanding and respond to adherence barriers. Cardiologists supervised message accuracy and clinical safety. | Usual care | MARS |  |
| Hartch  2024  USA | Surgeries | ≥18 years.;  smartphone;  at least one medication for a chronic disease;  Understand and speak English. | N=65; I=32; C=33; Dropout=I=2, C=2 (total 4; 6.2%). | The intervention used the Medisafe® mobile application, a medication adherence app that provided dose reminders, interactive checklists, and visual progress tracking to improve engagement in self-care. The app also included educational materials, peer-support features, and optional alerts for missed doses. Participants received app training and used it for 30 days to manage their prescribed therapies.  Nurses also performed follow-up calls to encourage consistent use and evaluated adherence data recorded in the app. The research team provided additional motivational support and ensured equitable access for low-income or low-digital-literacy participants. | Printed list of drugs | Adherence to Refills and Medications Scale (ARMS) |  |
| Babu  2024  India | hospital | >18 years; stroke within 1 month; modified Rankin scale (mRS) <5; at least one vascular risk factor: hypertension, diabetes, smoking, dyslipidemia; understanding and using Android smartphones  Mean age (SD)  I=60.3 (11.5); C=60.5 (10.2) | N=209; I=105; C=104; Dropout=I=2, C=2 (total 4; 1.9%). | The MaMoRS (Medication Adherence and Monitoring of Risk factors for Stroke) mobile app was designed for stroke survivors to support secondary prevention. The app provided personalized medication reminders, lifestyle tracking modules (for diet, exercise, and smoking), and real-time feedback on blood pressure and glucose readings.  It also offered educational content on stroke recovery, risk-factor control, and medication importance, with a secure system to send self-monitoring data to the study team. Delivered by a multidisciplinary stroke-care team. Neurologists, rehabilitation specialists, and nurses collectively trained patients in app use, monitored incoming health data, and provided feedback through teleconsultation. Nurses reinforced adherence behaviors, while clinicians reviewed flagged data and adjusted treatment plans as necessary. | Usual care | Morisky Medication Adherence Scale – 4 item |  |
| Ye  2024  China | Hospital | DM 2; hypertension; 45- 75 years; ability to use Wechat  Mean age (SD):  I = 51.2 (8.6); C = 52.4 (9.1)  Males: | N=174; Dropout=not reported. | Telehealth education delivered through the WeChat® platform for 26 weeks. Patients joined a WeChat group where they received daily educational posts (articles and videos) on diabetes and hypertension management, diet, exercise, medication adherence, and complication prevention. Interactive discussions allowed patients to ask questions, share experiences, and receive immediate clarification. Reminders for blood glucose monitoring, physical activity, and medication use were provided via messages and visual prompts. A multidisciplinary team including two endocrinologists, one cardiologist, and three nurses moderated the group. Nurses posted educational content daily, monitored participation, and provided real-time answers and feedback, while physicians guided clinical education, ensured accuracy of information, and reviewed patient queries requiring medical advice. | Usual care | SDSCA | 6 months |
| Xu  2024  China | hospitals | >18 years; atrial fibrillation (AF); Absence of dementia or other cognitive conditions that prevent the use of the app; Absence of valvular atrial fibrillation  Mean age (SD)  I=61.52 (9.96); C=61.77 (12.09)  Males n(%)  I=28(58.3); C=31(64.6) | N=209; I=57; C=56; Dropout=I=9, C=8 (total 17; 8.1%). | Alfalfa App, a mobile health platform designed for oral anticoagulation management. The app integrates patient education (10 video modules, 4–5 min each), remote medical consultation, automated medication and INR test reminders, and an anticoagulant community for peer interaction. It also includes an INR extreme-value warning system and provides blood pressure control advice. Patients report INR values, diet, and drug dosage through the app; doctors review submissions and provide feedback and dose adjustments remotely.  A team of experienced cardiologists and anticoagulation nurses oversaw the intervention | Usual care | Morisky Medication Adherence Scale | 3 months |
| Erdoğan  2024  Australia | Health Center | 18 years; diagnosis asthma for at least 1 year;  poor asthma control and using at least one asthma medication  Males n(%)  I=25 (12.5); C=31(31) | N=200; I=100; C=100; Dropout=I=9, C=8 (total 17; 8.5%). | A web-based education and counseling system designed to enhance self-management among adults with asthma. The program offered six weeks of interactive online sessions, including videos on correct inhaler use, breathing exercises, dietary guidance, and stress management modules. Participants accessed the platform via desktop or mobile device, completed quizzes to reinforce learning, and received weekly follow-up emails or text reminders encouraging module completion and adherence to prescribed therapy. Nurse educators facilitated virtual sessions, monitored log-in activity, and provided personalized online counseling for patients reporting difficulty managing asthma symptoms. | Usual care | MARS | 6 weeks |
| Lee 2024  China | Busan National University Hospital Diabetes Outpatient Clinic | 4069 years; DM2; HbA1c ≥ 7.0%; ability to use an app for selfmonitoring  Age: Medium (SD)  I=55.56 (8.92); C= 56.78 (8.30)  Males: n (%)  I= 21(48,8); C=44 (56.4) | N=121; I=43; C=78; Dropout=I=8, C=25 (total 33; 27.3%). | The APSC program was a mobile-based, automated, and individualized self-care support system for adults with type 2 diabetes. After baseline assessment, each participant received a personalized self-management plan on the mobile app that included daily health goal prompts, blood glucose monitoring reminders, and real-time feedback generated from user-entered data. The app analyzed trends and sent adaptive educational messages when glucose readings or dietary entries suggested nonadherence. Participants could also view progress charts and receive motivational messages to sustain engagement. The intervention ran for 18 months. Nurses trained in diabetes education coordinated the program, provided app training, and monitored adherence through the platform. They collaborated with endocrinologists and IT developers to refine personalized algorithms and feedback content. | Usual care | SDSCA |  |
| Hwang 2025  South Korea | Public health center and senior welfare center | Adults aged 65–80 years living alone with at least two chronic diseases | N=49; I=25; C=24; Dropout=I=4, C=3 (total 7; 14.3%) | An eight-week digital health coaching program delivered through a mobile application designed for older adults. The program included weekly educational videos on chronic disease self-management, assignments to test understanding, and individual telephone coaching sessions with nurses that involved goal setting and personalized feedback. Participants recorded daily medication use, diet, sleep and physical activity in the application. A wearable step-tracking band was used to record physical activity. The application also sent reminder messages and motivational feedback and allowed communication with coaches through an online discussion board | Usual chronic disease self-management without digital program | Korean version of the Self-Care of Chronic Illness Inventory; Morisky Medication Adherence Scale | 8 weeks |
| Kitsiou 2025  United States | University hospital heart failure clinic | Adults with stage C heart failure | N=27; I=13; C=14; Dropout=I=1, C=1 (total 2; 7.4%) | Participants used a mobile health system combining smartphone applications with connected monitoring devices including a body weight scale, blood pressure monitor and activity tracker. The system allowed daily monitoring of weight, blood pressure, symptoms, medication adherence and physical activity. The intervention included educational content on heart failure self-care and personalized motivational text messages aimed at improving disease knowledge, health beliefs and confidence in performing self-care behaviors | Usual heart failure care without digital monitoring | Self-Care of Heart Failure Index version 7.2 | 8 weeks |
| Lee 2025  South Korea | Neurology outpatient clinic of tertiary hospital | Adults ≥50 years with Parkinson disease | N=102; I=41; C=52; Dropout=I=6, C=3 (total 9; 8.8%) | Participants in the intervention group used a mobile application designed to monitor non-motor symptoms of Parkinson disease. The application allowed patients to record the presence and severity of symptoms such as pain, constipation, swallowing difficulties, sleep disturbance and fatigue using visual rating scales or pictorial representations. Participants were asked to record symptoms several times per week, and researchers monitored adherence and contacted participants if they stopped using the application regularly | Participants recorded the same symptoms using paper questionnaires instead of the mobile application | Self-Care of Chronic Illness Inventory; | 12 weeks |
| Lippke 2025  Germany | Recruitment through physicians and community outreach | Adults with cardiovascular disease including hypertension and heart failure | N=40; I=20; C=20; Dropout=I=2, C=1 (total 3; 7.5%) | Participants used a smartphone application designed to support cardiovascular disease self-management. The application included reminders for medication intake and physical activity, monitoring of blood pressure and physical activity, tools for goal setting and self-monitoring, feedback on health behaviors and educational information about cardiovascular risk factors. Data from activity trackers, smart watches and blood pressure monitors could be synchronized with the application | Standard care without mobile application | Self-Care of Hypertension Inventory (Maintainance) | 12 weeks |
| Meyer 2025  Germany | Recruitment through national health insurance provider | Adults with hypertension | N=102; I=52; C=50; Dropout=I=11, C=6 (total 17; 16.7%) | Participants received access to an internet-based digital therapeutic program that delivered structured sessions designed to support lifestyle change and hypertension self-management. The program included interactive educational modules about hypertension, breathing exercises aimed at activating the parasympathetic nervous system, behavioral goal setting, strategies for overcoming behavioral barriers, impulse control training, daily action planning and relapse prevention strategies. Participants could access audio exercises and written materials and were encouraged to use the program several times per week | Treatment as usual from general practitioners | Rief Adherence Index (medication adherence) | 3 months |
| Magnani 2025  United States | Cardiology clinics of a university hospital | Adults ≥21 years with atrial fibrillation receiving oral anticoagulant therapy | N=243; I=123; C=120; Dropout=I=15, C=17 (total 32; 13.2%) | Participants received a smartphone-based program with an animated conversational agent that simulated face-to-face dialogue with the patient. The digital agent delivered education about atrial fibrillation, medication adherence support, and guidance for recognizing symptoms and managing self-care problems. Participants were also provided with a portable heart rhythm monitoring device to check heart rhythm and rate. The system supported goal setting, monitoring of medication adherence, and reinforcement messages encouraging adherence to treatment and self-management behaviors | Smartphone with general health information application and heart rhythm monitor but without the conversational self-management program | Medication adherence measure | 12 months |
| Silberman 2025  United States | Decentralized recruitment through health insurance database | Adults aged 18–64 years with asthma | N=901; I=450; C=449; Dropout=I=107, C=88 (total 195; 21.7%) | Participants used a smartphone application connected to wearable devices, including a smartwatch and sleep monitor that recorded physiological parameters such as heart rate, respiratory rate and sleep data. The application prompted participants to log asthma symptoms and medication use, provided visual summaries of symptom patterns, and generated personalized notifications when deviations from baseline physiological data were detected. The system also provided educational information about asthma management and trigger avoidance, and encouraged regular symptom monitoring and engagement with the application | Modified application and monitoring devices without the self-management program features | Adherence to Refills and Medications Scale | 12 months |
| Yildirim Keskin 2025  Turkey | Cardiology outpatient clinic | Adults with hypertension receiving antihypertensive medication | **N=85; I=43; C=42; Dropout=I=3, C=2 (total 5; 5.9%)** | Participants received a **mobile application developed for hypertension monitoring and follow-up**. The application allowed patients to **enter daily blood pressure and pulse measurements**, record **medication intake**, and monitor **exercise and diet behaviors**. In the medication module patients indicated whether they had taken their medication and could select reasons for non-adherence. The application also sent **informational and motivational messages about hypertension management** and provided **graphical feedback on blood pressure trends over time**. Researchers monitored application use and sent reminder messages if data were not entered | Routine outpatient follow-up without mobile application | Hill-Bone Hypertension Treatment Compliance Scale | \| 6 weeks \| \| --- \| |

Legend: Summary of Diabetes SelfCare Activities (SDSCA); Medication Adherence Report Scale (MARS); New York Heart Association (NYHA)
