# Supplementary figures and images for: Effectiveness of Digital Health Interventions to Improve Self-Care in Patients With Chronic Diseases: Systematic Review and Meta-Analysis of Randomized Controlled Trials
Source: J Med Internet Res. 2026 Jun 9;28:e88708. doi: 10.2196/88708 (PMC13291736; doi:10.2196/88708)

# Supplementary File 4. Risk of bias

**4.1 Traffic-light plot**

**
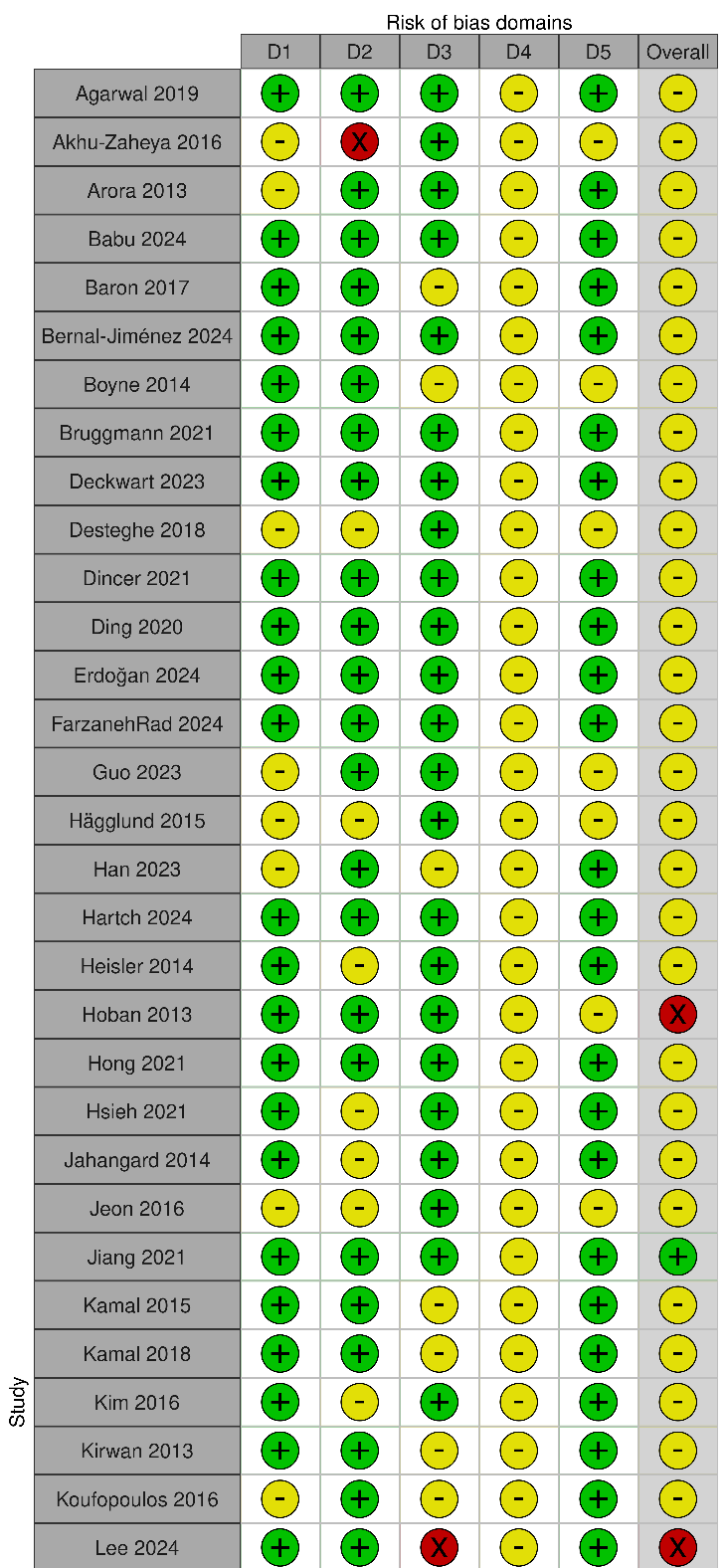

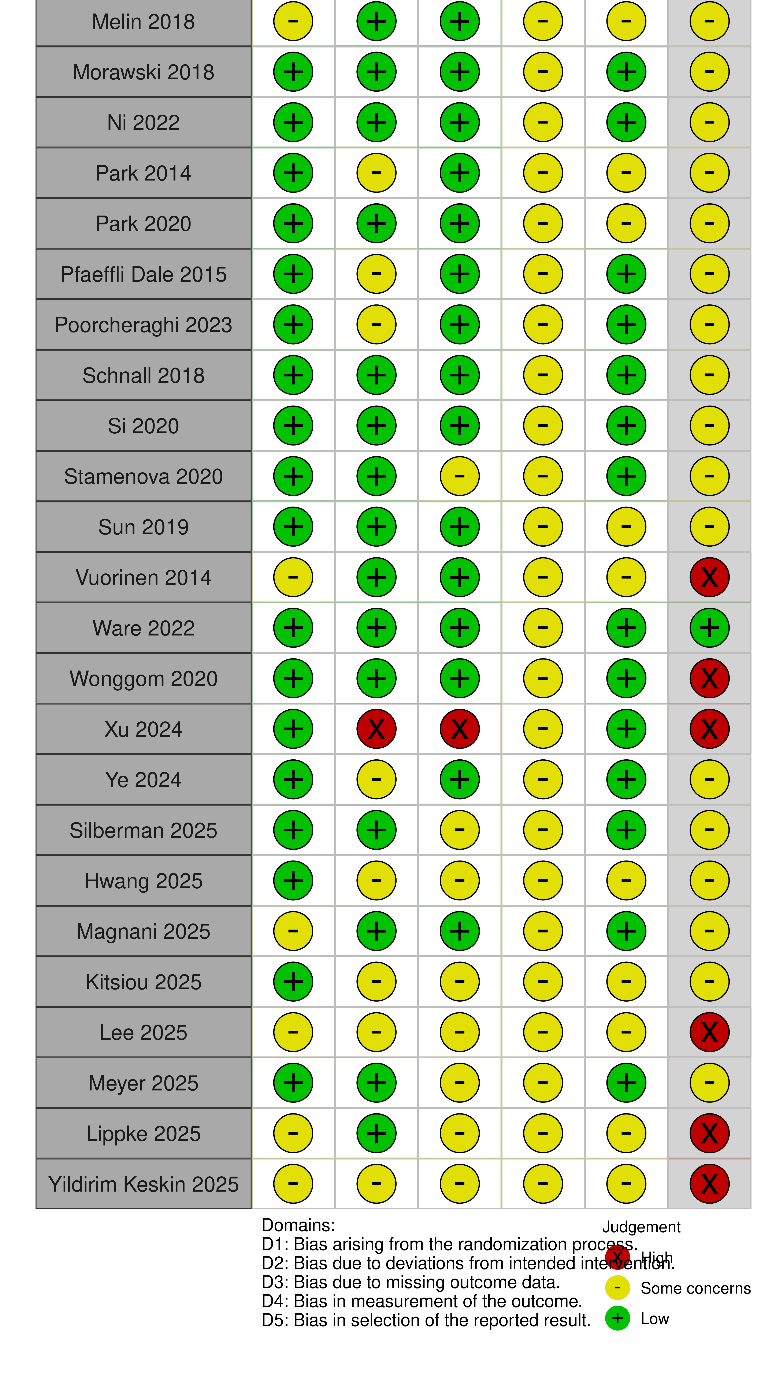
**

**4.2 Summary Plot**


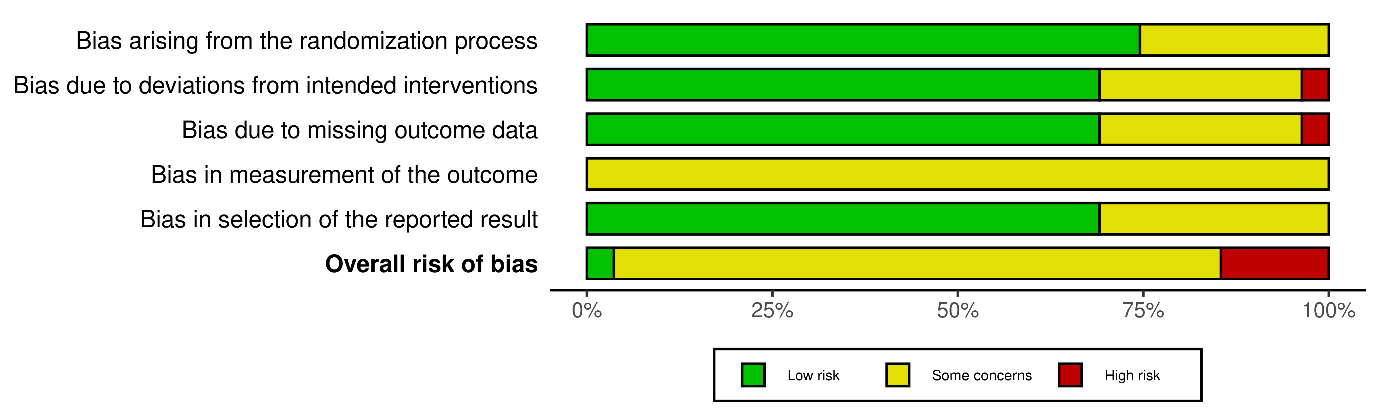

Supplement: Multimedia Appendix 4 [file jmir_v28i1e88708_app4.docx]
