# Supplementary material for: Effectiveness of Digital Health Interventions to Improve Self-Care in Patients With Chronic Diseases: Systematic Review and Meta-Analysis of Randomized Controlled Trials
Source: J Med Internet Res. 2026 Jun 9;28:e88708. doi: 10.2196/88708 (PMC13291736; doi:10.2196/88708)

# Supplementary File 5. Subgroup meta-analysis

5.1 SDSCA foot care


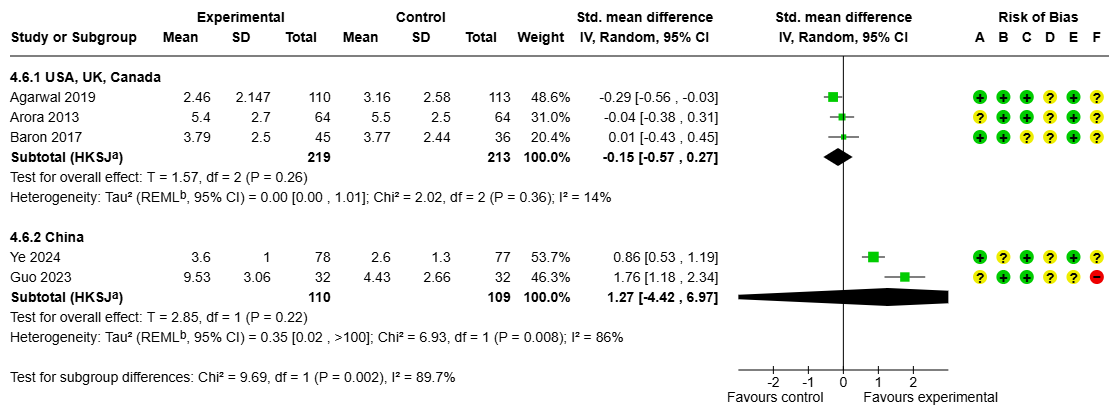


5.2 SDSCA exercise


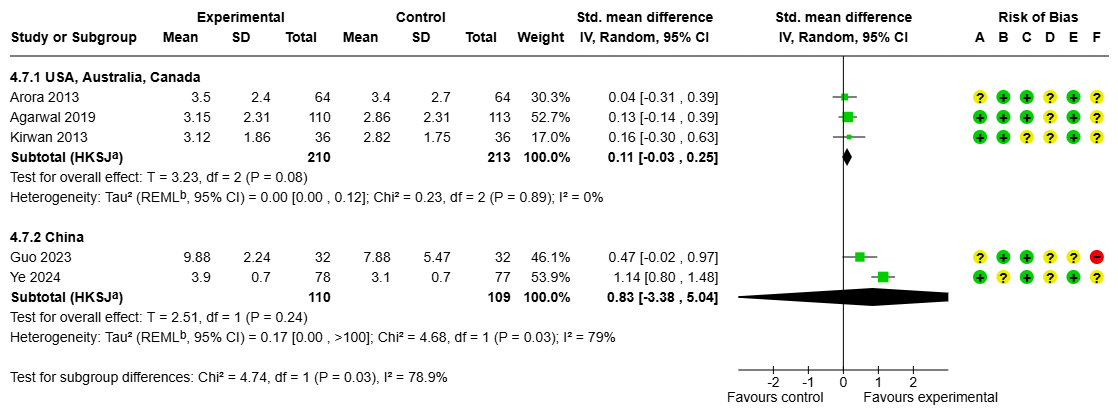


5.3 SDSCA glucose monitoring


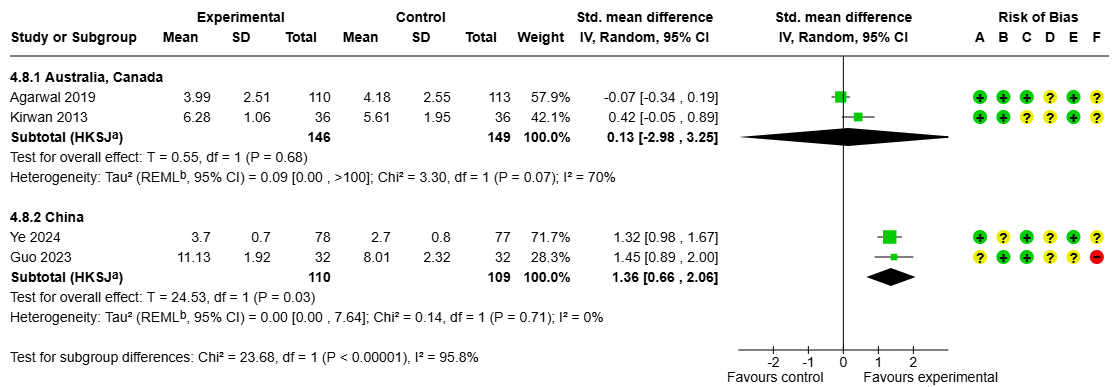


5.4 SCHFI Maintenance


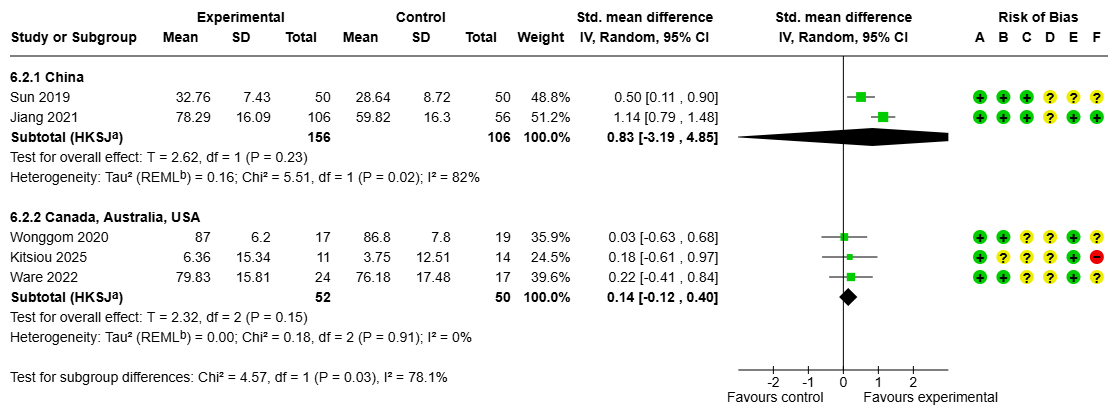


5.5 SCHFI Monitoring


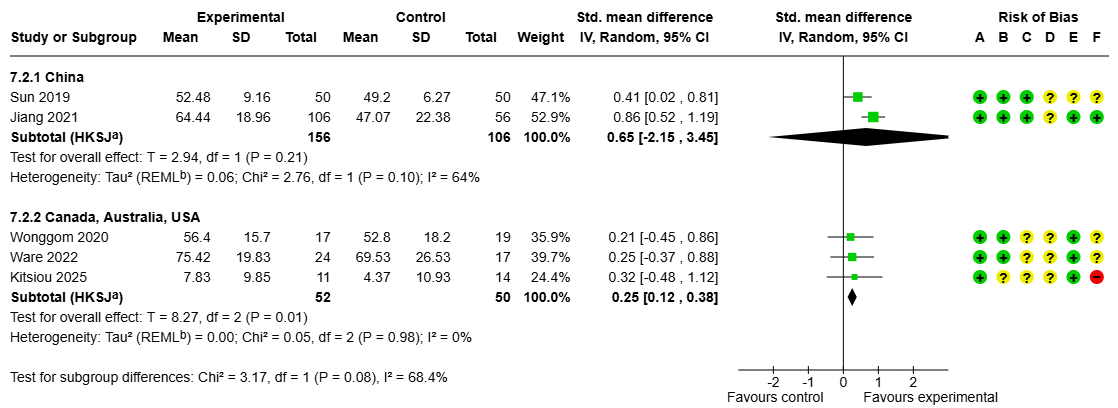


5.6 Medication adherence - Type of intervention


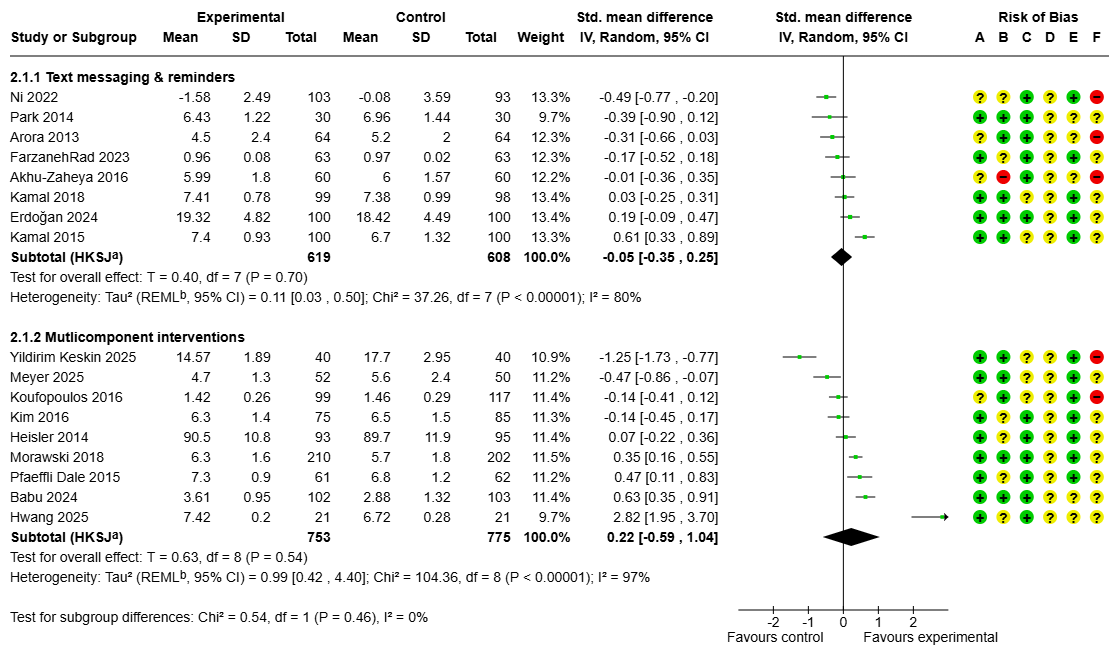


5.8 Medication adherence - Type of country


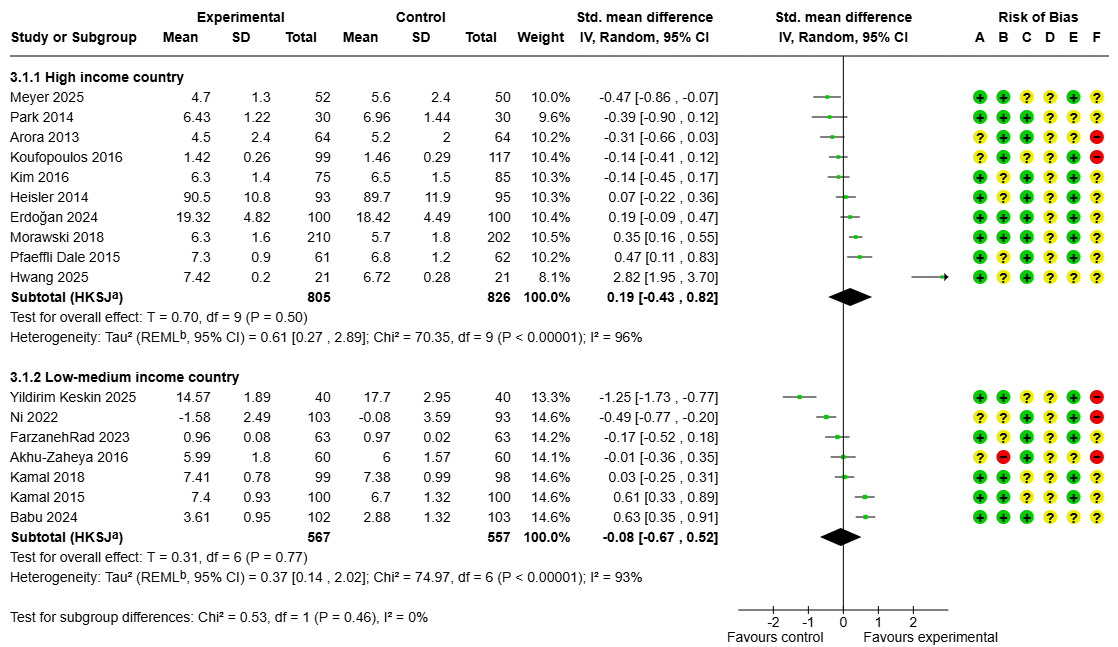

Supplement: Multimedia Appendix 5 [file jmir_v28i1e88708_app5.docx]
