# Supplementary material for: Effectiveness of Digital Health Interventions to Improve Self-Care in Patients With Chronic Diseases: Systematic Review and Meta-Analysis of Randomized Controlled Trials
Source: J Med Internet Res. 2026 Jun 9;28:e88708. doi: 10.2196/88708 (PMC13291736; doi:10.2196/88708)
Supplement: Multimedia Appendix 6 [file jmir_v28i1e88708_app6.docx]

**Supplementary File 6. Studies not included in the meta-analysis**

| **Author, year** | **Instrument** | **Results at the latest follow-up** |
| --- | --- | --- |
| Hoban, 2013 | SCHFI | At 60 days, greater physical activity, at 90 days, the difference was no longer significant. No other statistically significant difference |
| Hägglund, 2015 | EHFScB-9 item | Median (IQR): I=17(13, 22); C=21 (17, 25); p< 0.05 |
| Melin, 2018 | EHFScB-9 item | Median (IQR): C=23.5 (18.8-30.0); I=16.5 (12 - 22); p <0.05 |
| Desteghe, 2018 | MMAS-8 | Telemonitoring-based feedback: 7.8 (0.4); Observation phase: 7.6 (0.5); p = 0.003 |
| Xu, 2024 | MMAS-8 | Median (IQR): I=5.5 (4.5-6.8); C= 8 (7.0-8); p=<0.001 |
| Poorcheraghi, 2023 | MMAS-8 | N (%): Low: C=40 (43.48) I=22 (23.92); moderate: C=37(40.22) I=29(31.52); high: C=15 (16.3) I= 41 (44.56; p=<0.001 |
| Jahangard-Rafsanjani, 2015 | MMAS-8 | N (%): Low I= 11 (24) c= 20 (49); moderate/high I= 34 (76) c= 21 (51) p = 0.02 |
| Bernal-Jiménez, 2024 | Morisky Green and Levine Medication Adherence Questionnaire | N (%): I=54 (84); C=44(79); P=0.4 |
| Hsieh, 2021 | MARS | β=.606; 95% CI 0.253-0.96; P=.001) |
| Bruggmann, 2021 | ARMS | I=13.52, 95% CI 12.63-14.41; C=13.68, 95% CI 12.96-14.76; P=.33 |
| Hartch, 2024 | Medication adherence ARMS | Median (IQR): I= 14 (12.17); C=15(13,18); p=0.014 |
| Schnall, 2018 | CASE Adherence Index | β=-1.51; Standard error: 0.62; p= 0.017 |
| Si, 2020 | C-ESMS | Mean (SD): I= 47.1 (2.8); 40,7 (3,0); p <0.001 |
| Guo 2023 | SDSCA Diet | Mean (SD): C= 17.20 (5.47); I= 20.25 (4.82), p = .021 |
| Ye 2015 | SDSCA Diet | Mean (SD): C= 2.9 (0.7); I= 3.4 (0.9), p = .04 |
| Lee, 2024 | SDSCA Total score | Median (IQR): I=44.91 (12.01); C=41.34 (10.96); p=0.008 |
| Jahangard-Rafsanjani, 2015 | Diabetes Self-care Activity Measurement Scale | Median (IQR):   - General diet: I= 5.0(3-6); C= 0 (0-0); p = <.01 - Specific diet: I=2.0(1.5-3.25); c=2.5 (2-3.5); p = 0.33 - Exercise: I= 2.5 (1.5-3.5); C= 1.5 (0.25-3.5); p = 0.12 - Blood glucose monitoring: I=4.0(3-4); C= 2.0(0-3.25); p = <.01 - Foot care:I= 3.5 (2.25-4); c= 3.5(1.5-3.5); p = 0.02 |
| Dincer, 2021 | FSCBS | Median (IQR): I= 63 (30–75); C: 43 (25–75); p=0.045 |
| Ding, 2020 | Heart Failure Compliance Questionnaire | Mean (SD): I=0.06(0.49); C=-0.20 (1.03); p=0.04 |
| Boyne, 2014 | Heart Failure Compliance Questionnaire | Medium (SD); I=17.4 (4.5); C=20.8 (5.8); P= <0.001 |
| Jeon, 2016 | Self-care performance instrument | Mean (SD): I=91.11 (8.27); C = 86.58 (7.06); p= .001 |
| Nguyen, 2019 | CKD Self-Management Tool (CKD-SM) | Mean (SD): C=84.62 (15.36); I=102.74 (11.41); 95% CI 18,13 [13,14, 23,11] |
| Park, 2020 | The Alberto Chronic Obstructive Pulmonary Disease Self-Care Behavior Inventory | Mean (SD): I=122.32 ± 12.23; C=106.70 ± 18.4; p=0.05 |
| Stamenova, 2020 | Partners in Health (PIH) Scale | No statistically significant differences |
| Hong, 2021 | Partners in Health (PIH) Scale | Mean (SD): I= 87.67 (4.25); C=87.73 (7.03); p=<0.001 |
| Hwang, 2025 | Self-Care of Chronic Illness Inventory | \| Mean (SE): Self-care maintenance I=92.68 (1.56); C=87.89 (2.39); p=0.654. Self-care monitoring I=86.36 (3.76); C=76.02 (5.28); p=0.302. Self-care management I=83.00 (2.94); C=75.34 (3.78); p=0.753. \| \| --- \| |
| Lee, 2025 | Self-Care of Chronic Illness Inventory | Mean (SD): Self-care maintenance I=60.18 (10.34); C=57.31 (12.08); p=0.02. Self-care monitoring I=59.35 (13.85); C=56.92 (16.58); p=0.045. Self-care management I=44.96 (11.79); C=43.33 (12.78); p=0.26. |
| Lippke, 2025 | Self-Care of Hypertension Inventory - maintainance | Mean (SD):I=73.9 (16.0); C=54.1 (14.6); p<0.001. |
| Kitsiou 2025 | SCHFI management | Mean (SD): I=73.9 (20.09); C=73.64 (13.96); p=0.56. |
| Magnani 2025 | Self-reported non-adherence | No I=107 (87.0); C=103 (85.8). Yes I=16 (13.0); C=17 (14.2). Nonadherence OR (I vs C): 4 months 0.60 (0.30–1.21); 8 months 0.54 (0.26–1.10); 12 months 0.48 (0.19–1.21). |
| Silberman 2025 | Adherence to Refills and Medications Scale | “secondary PRO results at 12 months (eFigure 4 in Supplement 2) modestly  favored the DASM over the control group for self-reported medication adherence (standardized means difference [SMD], 0.28; 95% CI, 0.13-0.43; P < .001)” |
